# Neuroprotective effects of traditional Chinese medicine using zebrafish models ‐ a review

**DOI:** 10.3389/fphar.2026.1810881

**Published:** 2026-05-25

**Authors:** Jun Shao, Yun An, Ruicong Ding, Shanshan Wang, Xueke Wang

**Affiliations:** 1 Henan Province Hospital of Traditional Chinese Medicine (The Second Affiliated Hospital of Henan University of Chinese Medicine), Zhengzhou, China; 2 Shandong University of Traditional Chinese Medicine, Jinan, China; 3 Henan Integrative Medicine Hospital, Zhengzhou, China

**Keywords:** clinical translation, drug screening, neurological disorders, neuroprotection, Traditional Chinese Medicine, zebrafish

## Abstract

Neurological disorders, including Alzheimer’s disease, Parkinson’s disease, and stroke, remain major causes of global disability and mortality, with limited neuroprotective therapies available. Traditional Chinese medicine (TCM) offers multi-target therapeutic potential, but its mechanistic complexity requires systematic investigation using appropriate model systems. Zebrafish (*Danio rerio*) has emerged as a valuable vertebrate platform for TCM neuroprotection research due to its genetic homology with humans, optical transparency, and high-throughput screening compatibility. This review summarizes the application of zebrafish models in studying TCM for Alzheimer’s disease, Parkinson’s disease, cerebral ischemia, epilepsy, insomnia, depression, and spinal cord injury. Key findings indicate that TCM metabolites exert neuroprotective effects through multiple mechanisms, including anti-oxidative stress, anti-neuroinflammation, anti-apoptosis, neurotransmitter modulation, neurogenesis promotion, and vascular protection. Zebrafish models have proven particularly useful for high-throughput screening of active metabolites, real-time *in vivo* imaging of neurovascular processes, and rapid safety assessment. However, limitations such as the absence of a layered neocortex, differences in drug metabolism, and the predominantly acute nature of current models must be acknowledged. Addressing these challenges through model standardization, multi-omics integration, and cross-species validation will further enhance the translational relevance of zebrafish-based TCM research. This review provides a practical framework for leveraging zebrafish models to advance the mechanistic understanding and clinical development of neuroprotective TCM therapies.

## Graphical Abstract

**Figure:**
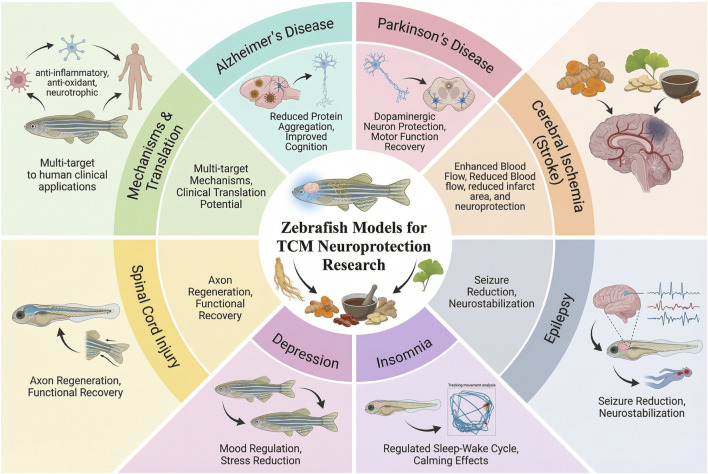

## 1 Introduction

### 1.1 Global challenges of neurological diseases

Neurological disorders, which encompass neurodegenerative diseases (e.g., Alzheimer’s disease, Parkinson’s disease), cerebrovascular diseases (e.g., ischemic stroke), neurodevelopmental disorders, and neuropsychiatric disorders, represent a significant challenge to global public health in the 21st century (Ma H. et al., 2025). The World Health Organization reports that neurological disorders are the primary cause of disability-adjusted life years (DALYs) worldwide, and the socioeconomic burden is steadily increasing (Qin et al., 2025). Current treatments for these diseases primarily aim at symptomatic relief, while neuroprotective therapies capable of effectively halting or reversing neuronal damage and death have yet to yield significant breakthroughs (Li H. et al., 2025). The pathophysiological mechanisms underlying neurological diseases are highly complex, involving multiple factors such as genetics, environment, immunity, and metabolism. Consequently, achieving the desired outcomes with single-target drugs is often challenging, resulting in prolonged research and development timelines, elevated costs, and high failure rates (George et al., 2022; Gong et al., 2018). Therefore, the exploration of new research models and the development of multi-target, multi-pathway therapeutic strategies have emerged as urgent priorities in the fields of neuroscience and pharmacology.

### 1.2 Advantages of the zebrafish model

**FIGURE 1:**
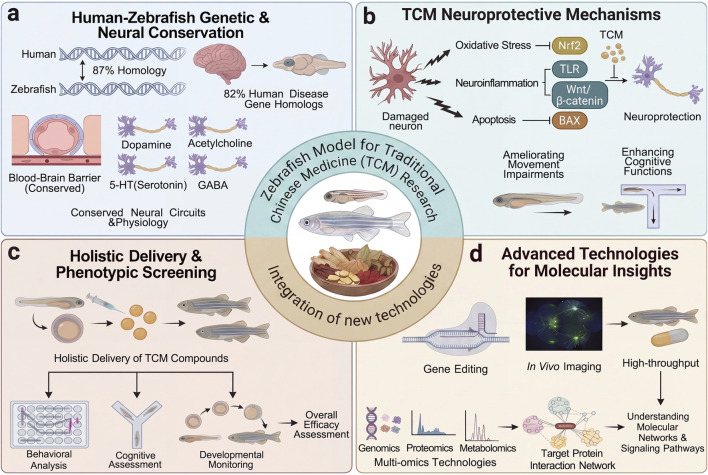
A schematic overview of integrated research system for neuroprotective effects of TCM utilizing zebrafish. **(a)** Human-Zebrafish Genetic and Neural Conservation: Zebrafish exhibit 87% genetic homology with humans, and approximately 82% of human disease-associated genes have orthologs in zebrafish. This evolutionary conservation extends to the structural and functional preservation of the blood-brain barrier, core neurotransmitter systems and neural circuitry, establishing zebrafish as a valid translational model for human neurological research. **(b)** TCM Neuroprotective Mechanisms: When neurons face damage, TCM steps in as a multi-targeted defender: it calms oxidative stress and neuroinflammation via the Nrf2 and TLR pathways, blocks cell death through BAX inhibition, and repairs neural function by activating Wnt/β-catenin signaling. The results are shielded neurons, smoother movement, and sharper cognitive performance. **(c)** Holistic Delivery and Phenotypic Screening: This section describes the holistic administration of complex TCM formulations in zebrafish, followed by comprehensive phenotypic assessment. Integrated endpoints include behavioral analysis, cognitive function evaluation, and developmental monitoring, enabling the systematic characterization of TCM’s overall therapeutic efficacy and its synergistic biological effects. **(d)** Advanced Technologies for Molecular Insights: Cutting-edge technologies, including CRISPR/Cas9-mediated gene editing, *in vivo* fluorescence imaging, high-throughput screening, and multi-omics approaches (genomics, proteomics, metabolomics), are employed to dissect the molecular underpinnings of TCM action. These tools facilitate the mapping of target protein interaction networks and signaling pathways, thereby elucidating the precise molecular mechanisms of TCM-induced neuroprotection.

In the quest for more efficient preclinical research models with enhanced translational potential, zebrafish have swiftly emerged as a novel vertebrate model over the past two decades. Their primary advantage lies in the ability to balance biological complexity with the practicality of high-throughput experiments, thereby offering a distinctive perspective for addressing intricate challenges in neuroscience (Kalueff et al., 2014). Zebrafish share a high degree of genetic similarity, functional homology, physiological, and developmental processes with humans (Zizioli et al., 2023) (Figure 1). The zebrafish genome has over 87% homology with the human genome (Zheng et al., 2025). Furthermore, 82% of all human disease-related genes are present as direct homologs in zebrafish (Shankar et al., 2021). Regarding brain anatomy, zebrafish and humans share a conserved vertebrate brain plan, including the telencephalon, diencephalon, midbrain, cerebellum, and spinal cord. Key regions relevant to neurological diseases are evolutionarily conserved: the dorsolateral pallium (Dl) is homologous to the mammalian hippocampus, the dorsomedial pallium (Dm) to the amygdala, and the ventral telencephalic areas to the basal ganglia. The major neurotransmitter systems (e.g., dopamine, acetylcholine, 5-hydroxytryptamine, GABA) and the blood-brain barrier are also highly conserved, providing a robust biological foundation for modeling human neurological disorders (Howe et al., 2013; Lieschke and Currie, 2007; MacRae and Peterson, 2015). However, important differences exist. The zebrafish telencephalon develops through eversion, whereas the mammalian telencephalon undergoes evagination. Zebrafish lack a six-layered neocortex; their dorsal pallium is organized primarily as nuclei rather than laminated structures (Ito and Yamamoto, 2009; Mueller and Wullimann, 2009). The total number of neurons in the zebrafish central nervous system is approximately 100,000 at the larval stage and 10 million in the adult, which is several orders of magnitude lower than the approximately 86 billion neurons in the human brain (Azevedo et al., 2009; Herculano-Houzel, 2009). Furthermore, the postsynaptic density (PSD) proteome of zebrafish has been shown to be less complex than that of mammals (Bayés et al., 2017). These differences mean that zebrafish cannot fully recapitulate higher-order cognitive dysfunctions, but they are well-suited for studying fundamental neuropathological mechanisms and for high-throughput drug screening (Imberechts et al., 2025). Furthermore, zebrafish have been increasingly recognized as a valuable model for nutritional neuroscience and brain disorders, allowing investigation of how dietary factors modulate neuropathological processes (Rafique et al., 2023).

Zebrafish offer several experimental advantages due to their small size, low rearing costs, high reproductive capacity (producing hundreds of embryos weekly) and rapid embryonic development (Kraus et al., 2022). Their suitability for *in vitro* fertilization and whole-body transparency enables researchers to conduct large-scale, non-invasive, *in vivo*, real-time imaging. Zebrafish allow for the observation of neural development, neuronal apoptosis, angiogenesis, and drug responses at single-cell resolution throughout the life cycle, particularly during early developmental stages and other dynamic processes (Lieschke and Currie, 2007; Zon and Peterson, 2005). Zebrafish larvae, due to their small size and quick growth, are an excellent choice for high-throughput screening applications. They can be easily accommodated in 96- or 384-well plates, facilitating large-scale compound screening, toxicity testing, and behavioral analysis. Integration with automated imaging and liquid handling systems further enhances the zebrafish model’s capacity for rapid identification of potential drug candidates and initial evaluations of efficacy and safety. This streamlined approach significantly reduces the duration of the initial phases of drug discovery (Chakravarthy et al., 2014; Li et al., 2026; Zon and Peterson, 2005).

### 1.3 The role of Traditional Chinese Medicine (TCM) in neuroprotection

Traditional Chinese Medicine (TCM) boasts a rich history spanning thousands of years in the treatment of neurological disorders, including stroke, dementia, and epilepsy, along with a substantial body of clinical experience. The efficacy of TCM is rooted in its holistic approach, which regulates bodily functions through the synergistic use of multiple botanical drugs. An increasing number of studies have demonstrated that various monomers, active metabolites, and compound formulas in Chinese medicine, such as ginsenosides, baicalin, tanshinone, and Liuwei Dihuang Pill, exhibit significant neuroprotective properties (Lv et al., 2025; Song et al., 2022; Wang et al., 2016; Yuan et al., 2023; Zhu et al., 2021).

The “multi-component, multi-target, multi-pathway” characteristics of Chinese medicine complicate the investigation of its material basis and mechanisms of action. Traditional *in vitro* research methods, which focus on a single target or cell line, struggle to elucidate the comprehensive regulatory mechanisms involved. This limitation hinders the modernization and advancement of TCM (Huang et al., 2020; Li J. et al., 2020). Zebrafish model, as a comprehensive living system, offers a robust platform for advancing research on Chinese medicine. It not only facilitates the holistic delivery of the intricate metabolites of TCM compounds but also enables the intuitive assessment of their overall efficacy through phenotypic screening, such as ameliorating movement impairments and enhancing cognitive functions. Furthermore, by integrating gene editing, *in vivo* imaging, and multi-omics technologies, scientists can methodically investigate the molecular networks and signaling pathways underlying the neuroprotective effects of TCM in zebrafish models. This approach enables a transition from mere identification to understanding the mechanisms at play, thereby enhancing the depth of knowledge in this field (Chen, et al., 2020; Huang et al., 2020; Li X. et al., 2025; MacRae and Peterson, 2015).

This review systematically organizes the findings related to the application of the zebrafish model in TCM neuroprotection research in recent years. Its aim is to provide theoretical references and strategic guidance for the future advancement of this interdisciplinary field.

## 2 Zebrafish neurological disease models

**FIGURE 2:**
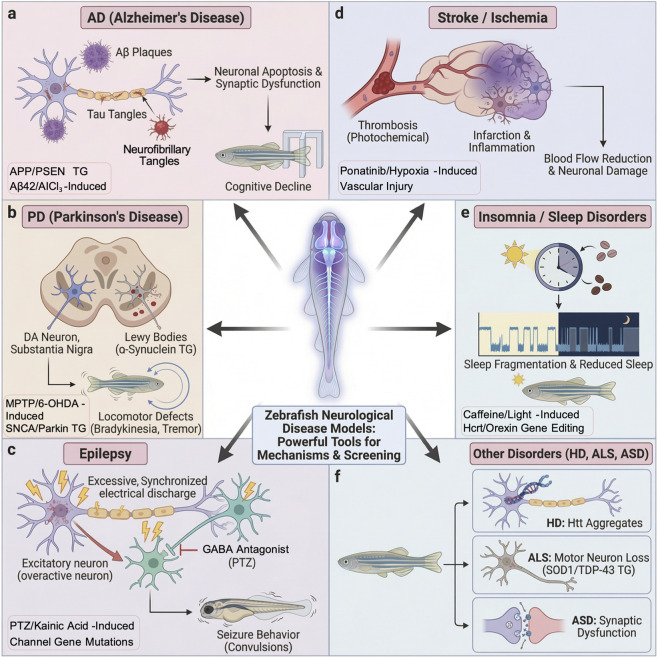
Establishment and pathological characteristics of zebrafish models for major neurological disorders. **(a)** AD models are generated via APP/PSEN transgenesis or Aβ42/AlCl_3_ administration, recapitulating Aβ plaque deposition, neurofibrillary tangle formation, synaptic dysfunction, and cognitive decline. **(b)** PD models are induced by MPTP/6-OHDA exposure or *SNCA/Parkin* genetic modification, exhibiting dopaminergic neuron loss in the substantia nigra, Lewy body formation, and locomotor deficits including bradykinesia and tremor. **(c)** Epilepsy models are established via PTZ/kainic acid treatment or ion channel gene mutations, characterized by excessive synchronized electrical discharges in overactive excitatory neurons, impaired GABAergic inhibition, and seizure-like convulsive behavior. **(d)** Stroke/ischemia models are constructed through photochemical thrombosis or ponatinib/hypoxia-induced vascular injury, mimicking thrombosis, infarction, inflammation, reduced cerebral blood flow, and subsequent neuronal damage. **(e)** Insomnia/sleep disorder models are generated by caffeine/light stimulation or Hcrt/orexin gene editing, displaying sleep fragmentation and reduced total sleep duration to facilitate sleep mechanism studies. **(f)** Models of other neurological disorders, including Huntington’s disease (HD, with mutant Htt aggregation), amyotrophic lateral sclerosis (ALS, with motor neuron loss via SOD1/TDP-43 modification), and autism spectrum disorder (ASD, with synaptic dysfunction), are established via transgenic or gene-editing approaches.

Zebrafish have emerged as a potent model for simulating human neurological disorders. By employing genetic techniques such as knockout/knock-in and transgenesis, along with chemical methods like neurotoxin exposure, researchers have effectively replicated various fundamental pathological characteristics in zebrafish (Figure 2). This progress has established a basis for conducting drug screening and exploring mechanisms (Dash and Patnaik, 2023; Liu, 2023; Pansera et al., 2025).

### 2.1 Models of Alzheimer’s disease (AD)

Alzheimer’s disease (AD) is the most prevalent neurodegenerative disorder, characterized primarily by age-related deposits of β-amyloid (Aβ) and neurofibrillary tangles resulting from the hyperphosphorylation of Tau protein (Rafique et al., 2025). Zebrafish models of AD are primarily classified into two categories: transgenic models and chemically induced models (Freeman and Kiper, 2022; Wang K. et al., 2021). In recent years, with the advancement of genetic engineering technologies such as CRISPR/Cas9 and the deepening of AD pathogenesis research, zebrafish models have been further optimized and expanded, playing an increasingly important role in AD mechanism exploration, drug screening, and early pathological stage research (Dey et al., 2024; Saleem and Kannan, 2018).

#### 2.1.1 Transgenic models

The APP/PSEN transgenic model involved the targeted expression of mutant human amyloid precursor protein (APP) and presenilin (PSEN1/2) in zebrafish neurons. This approach led to the overproduction and aggregation of Aβ, resulting in age-related neuronal apoptosis, synaptic dysfunction, and AD-like phenotypes, including a decline in learning and memory abilities (Kotova et al., 2025; Yenkoyan et al., 2025). A transgenic zebrafish model with overexpressed human mutant Tau proteins (e.g., P301L, A152T) demonstrates hyperphosphorylation, aggregation, and neuronal death, offering a tangible representation to elucidate the molecular underpinnings of Tau protein pathologies (Tauopathies) (Barbereau et al., 2020; Lopez et al., 2017).

The application of CRISPR/Cas9 gene editing technology has significantly expanded the capabilities of zebrafish AD modeling, enabling the generation of knock-in models that more closely recapitulate human genetic conditions (Lardelli et al., 2024; Kroll et al., 2025). A study has developed multiple zebrafish strains carrying early-onset familial AD (EOfAD)-like and non-EOfAD-like mutations in orthologs of human *PSEN1, PSEN2,* and *SORL1* genes, an approach that enables systematic transcriptomic profiling of early-stage AD-associated pathological alterations (Lardelli et al., 2024). Using CRISPR/Cas9-mediated genome editing, researchers have established F0 knockout mutants of four late-onset AD (LOAD) susceptibility genes (*sorl1, abca7, trem2, and cd2ap*); this strategy has facilitated the delineation of perturbed signaling cascades and the identification of betamethasone as a potential therapeutic candidate capable of restoring normal sleep phenotypes in *psen2* knockout larvae (Kroll et al., 2025).

Beyond gene knockout and knock-in strategies, the zebrafish model has been used to study APOE4, the main genetic risk factor for late-onset AD. Embryos treated with a recombinant amino-terminal fragment of human ApoE4 (nApoE4^1−151^) showed increased mortality, developmental abnormalities, and reduced pigmentation, unlike those treated with the ApoE3 variant. The nApoE4^1−151^ fragment translocated to neuronal nuclei and induced tau pathology (increased PHF-1 immunoreactivity at Ser396/Ser404). These findings support the toxic-gain-of-function hypothesis of ApoE4 and establish a rapid zebrafish platform for drug screening (McCarthy et al., 2022). Furthermore, commercialized technical platforms currently enable the rapid generation of stable transgenic lines expressing AD-related mutations in APP, tau, or presenilin genes through CRISPR-based genome editing or transgenesis strategies (Sang et al., 2025). Comprehensive review studies have systematically summarized these emerging zebrafish AD models, highlighting their distinct advantages and intrinsic limitations in elucidating the molecular mechanisms underlying tauopathy and AD pathogenesis (Lopes and Guil-Guerrero, 2025; Yenkoyan et al., 2025).

#### 2.1.2 Chemically induced models

The Aβ42 peptide injection model involves the direct injection of synthetic Aβ42 peptide or Aβ_1-42_ peptide into the ventricles or specific brain regions of zebrafish. This method acutely induces neuroinflammation, oxidative stress, and cognitive dysfunction, rendering it appropriate for swiftly screening drugs that can inhibit Aβ toxicity (Bhattarai et al., 2017; Bhattarai et al., 2020; Nery et al., 2014). Prolonged exposure to specific metal ions (e.g., aluminum chloride (AlCl_3_)) or substances (e.g., D-galactose, scopolamine, okadaic acid (OKA), streptozotocin (STZ)) can trigger AD-like pathological alterations in zebrafish, such as Aβ accumulation, Tau protein phosphorylation, cholinergic neuron impairment, and cognitive decline. These models offer cost-effective options and are conducive to extensive screening (Amoah et al., 2023; Nada et al., 2016; Koehler et al., 2019; Luo et al., 2024; Rafique et al., 2023; Sang et al., 2020).

Recent refinements to chemically induced zebrafish models of AD have further enhanced their utility in pathogenesis research and preclinical drug discovery. A rapid, cost-effective larval model was established via co-treatment with AlCl_3_ and D-galactose for 72 h, which induces learning/memory deficits, Aβ_1-42_ deposition, and elevated acetylcholinesterase activity—rendering it suitable for high-throughput screening (Luo et al., 2024). AlCl_3_ and OKA have been validated as effective inducers of AD-like cognitive dysfunction, using an optimized induction protocol (Raduan et al., 2025). A chronic AlCl_3_-induced model recapitulates oxidative stress, cholinergic dysfunction, neurodegeneration, and gut-brain axis alterations, and facilitates the investigation of sex-specific susceptibility to AD (Vaja et al., 2025). Additionally, a STZ-induced sporadic AD model has been established; intracerebroventricular administration of STZ recapitulates Aβ deposition, tau hyperphosphorylation, neuronal loss, and cognitive deficits within 7 days (Dhiman et al., 2025). Scopolamine-induced cognitive impairment models remain widely used for screening natural neuroprotective agents; a recent review compiled 21 studies evaluating 28 natural substances against scopolamine-induced AD-like behaviors in zebrafish (Abidar et al., 2025).

### 2.2 Parkinson’s disease (PD) modeling

The typical pathology of Parkinson’s disease (PD) involves the gradual degeneration of dopaminergic (DA) neurons in the substantia nigra of the midbrain, resulting in motor manifestations like resting tremor and bradykinesia. Zebrafish exhibit a dopamine system that closely resembles that of mammals, rendering them a valuable model for investigating PD (Dodiya et al., 2025; Doyle and Croll, 2022).

#### 2.2.1 Neurotoxin modeling

The MPTP/MPP^+^ model involves the administration of 1-methyl-4-phenyl-1,2,3,6-tetrahydropyridine (MPTP) and its active metabolite MPP^+^, which are well-established neurotoxins causing selective degeneration of DA neurons. Exposure of zebrafish to these compounds through immersion or injection leads to progressive loss of DA neurons, impaired locomotor function (e.g., reduced swimming speed, increased circling behavior), and downregulation of relevant genes such as *dat* and *th*(Chen A. et al., 2016; Li et al., 2022). Recently, refinements have been made to the intraperitoneal injection model using MPTP in adult zebrafish, revealing that a single dose of 100 μg/g of MPTP effectively induces PD-like symptoms on days 3–5, offering a specific timeframe for assessing therapeutic interventions (Omar et al., 2023). 6-Hydroxydopamine (6-OHDA) and rotenone models are equally adept at selectively harming DA neurons. These models have been extensively employed in zebrafish to replicate the pathological progression of PD induced by various causes (Briñez-Gallego et al., 2023; Wasel and Freeman, 2020).

In addition to the above-mentioned neurotoxins, paraquat—a widely used herbicide known to increase PD risk by dysregulating dopaminergic systems in humans—has also been employed to induce PD-like phenotypes in zebrafish (Mohamad Najib et al., 2023). Short-term paraquat exposure reduces motor activity, dysregulates cholinergic and serotonergic systems, and inhibits tyrosine hydroxylase activity (Kim et al., 2022). Standardized induction protocols are available (Harini et al., 2024). Mechanistically, paraquat downregulates dopamine-related genes (*dat, th1*), upregulates pro-inflammatory genes (*il-1α, il-1β, tnf-α, cox-2*), and causes dopaminergic neuron loss, effects reversible by metallothionein 2 (Mohamad Najib et al., 2023). Paraquat also alters purinergic signaling in the adult zebrafish brain (Bortolotto et al., 2025) and exerts ototoxicity on neuromast hair cells (Tantry and Santhakumar, 2024). Collectively, paraquat serves as a valuable chemical inducer for PD modeling and neuroprotective screening.

#### 2.2.2 Transgenic models

Although zebrafish do not possess the α-synuclein (*SNCA*) gene, the expression of human wild-type or mutant *SNCA* through transgenic technology can replicate the formation of Lewy bodies in zebrafish, resulting in the progressive degeneration of DA neurons (Zini et al., 2025). A stable transgenic line expressing mCherry-tagged human α-synuclein has been generated, recapitulating key PD phenotypic traits during the larval stage. Beyond α-synuclein, zebrafish have been extensively utilized to model mutations in other familial PD-associated genes. The modeling of mutations or knockouts in genes linked to familial PD, such as *LRRK2, PINK1,* and *Parkin*, serves as a valuable tool for investigating the genetic susceptibility and pathogenesis of PD (Bangeppagari et al., 2025; Fett et al., 2010; Seegobin et al., 2020).

Knockdown of *LRRK2* has been shown to cause neuronal loss, synuclein aggregation, and developmental perturbations, including axis curvature and ocular abnormalities (Prabhudesai et al., 2016). Loss of *PINK1* function has been demonstrated to impair development, reduce dopaminergic neuron populations, and induce neurodegeneration, with distinct groups of dopaminergic neurons exhibiting differential sensitivity to *Pink1* deficiency (Anichtchik et al., 2008; Flinn et al., 2013). The zebrafish Parkin protein shares 62% identity with its human counterpart (78% in functionally relevant regions), and parkin-deficient zebrafish have been established as a vertebrate model for early-onset Parkinson’s disease, recapitulating complex I deficiency and dopaminergic neuron loss (Flinn et al., 2009). More recently, a stable *PARK7* knockout zebrafish line has been developed, representing the first stable genetic zebrafish model of PD exhibiting both motor and non-motor symptoms alongside a reduction in dopaminergic neurons at the larval stage (Solheim et al., 2026). Additionally, *Atp13a2*-deficient zebrafish have been generated, demonstrating degeneration of dopaminergic neurons, lysosomal dysfunction, and impairment of intracellular trafficking (Nyuzuki et al., 2020). Together, these transgenic models serve as valuable tools for investigating the genetic susceptibility and pathogenesis of PD, as well as for high-throughput screening of disease-modifying compounds (Dodiya et al., 2025).

### 2.3 Cerebral ischemia model

Zebrafish, with their robust regenerative capacity, transparent vasculature, and high hypoxia tolerance, are a unique model for studying stroke-induced neurological and vascular injury and repair (Chen J. et al., 2024; Crilly et al., 2022; Meng et al., 2025). Established stroke models enable investigation of post-stroke brain injury and repair via behavioral assays, *in vivo* imaging, and drug screening (Chen J. et al., 2021).

Chemical hypoxia models (e.g., sodium nitroprusside, nitrogen gas, or sodium sulfite) induce global cerebral ischemia, causing neuronal death and behavioral deficits, and are well suited for large-scale anti-hypoxic drug screening (Marino et al., 2020). A refined hypoxia-reoxygenation platform recapitulates neonatal hypoxic-ischemic encephalopathy (HIE) features—including reduced survival, motor impairment, neuronal loss, vasoconstriction, and decreased cerebral blood flow—enabling mechanistic studies and therapeutic validation (Lin et al., 2025). Photochemical/laser-induced models use photosensitizers (e.g., Rose Bengal) and laser irradiation to generate localized thrombi and ischemic foci (Lee et al., 2016; Meng et al., 2025). Real-time imaging allows observation of thrombus formation, blood flow changes, neuronal damage, and blood-brain barrier disruption, offering a dynamic platform for evaluating thrombolytic and neuroprotective agents (Hwang et al., 2023; Nayaka et al., 2025). Using this model, age-dependent differences in post-stroke recovery have been revealed: young zebrafish restore cerebral blood flow by 7 days and learning ability by 14 days, whereas aged zebrafish show prolonged functional recovery despite similar hemodynamic restoration (Mizoguchi et al., 2025).

Drug-induced cerebral ischemia models have also been developed. Ponatinib induces a cerebral ischemia model in zebrafish by inhibiting PDGFR, successfully mimicking key cellular and molecular aspects of human ischemic stroke, including cerebrovascular endothelial damage, thrombosis, reduced blood flow, inflammation, and apoptosis (Zhu et al., 2020). Phenylhydrazine (PHZ), an oxidative hemolysis inducer, has been used to establish a thrombosis-based cerebral ischemia model, where peripheral and cerebral blood flow are quantified in fluorescence-labeled larvae for anti-stroke drug screening (Wang Q. et al., 2022). Additionally, transgenic models such as Tg (kdrl:DenNTR) enable nitroreductase (NTR)-mediated endothelial cell ablation to simulate vascular injury (Chen W. et al., 2021; Meng et al., 2025). Recent advances have enhanced drug screening and understanding of ischemic injury mechanisms (Li L. et al., 2024; Lin et al., 2023; Zhang C. et al., 2025). A comprehensive review details zebrafish-based screening for ischemic stroke therapeutics, covering experimental design factors (developmental stage, strain, administration routes, induction methods) (Nayaka et al., 2025).

It should be noted, however, that zebrafish are less suitable for modeling transient ischemic attack (TIA) due to the absence of established protocols for reversible, short-duration focal occlusion, as well as for modeling large-vessel occlusion subtypes such as middle cerebral artery occlusion (MCAO), given the significant anatomical differences in cerebral vasculature between zebrafish and mammals. Thus, the zebrafish model is best positioned as a complementary platform for high-throughput screening and mechanistic studies, with findings requiring subsequent validation in mammalian models (Meng et al., 2025; Mizoguchi et al., 2025).

### 2.4 Epilepsy modeling

Epilepsy is a neurological disorder resulting from aberrant, excessively synchronized neuronal discharges within the brain (Tóth et al., 2017). Zebrafish larvae, with their swift neurological maturation and susceptibility to chemical triggers, represent an optimal model for conducting high-capacity screenings of antiepileptic drugs (Burrows et al., 2020). A chemically induced model using pentylenetetrazol (PTZ), a GABA_A_ receptor antagonist, can reliably elicit typical epileptic-like behaviors (e.g., convulsions, frenzied swimming) in zebrafish larvae through immersion treatment (Moradi-Afrapoli et al., 2017). This model is extensively employed for screening and assessing anticonvulsant drugs (Afrikanova et al., 2013). In recent years, the PTZ zebrafish model has continued to be widely used for evaluating novel anticonvulsant candidates (Fernández et al., 2025; Ciubotaru et al., 2025). Furthermore, alternative convulsants like erythrocyanine (kainic acid) have been utilized to simulate epilepsy through distinct mechanisms (D'Amora et al., 2023; Heylen et al., 2021).

Beyond chemical induction, the advent of gene editing technologies, particularly CRISPR/Cas9, has facilitated the rapid development of zebrafish models for gene mutations linked to human epilepsy (Grone et al., 2016). A landmark study generated loss-of-function models for 48 candidate epilepsy genes and identified five genes (*arfgef1*, *kcnd2*, *kcnv1*, *ubr5*, and *wnt8b*) that exhibit seizure-like behavior and hyperexcitability, providing robust *in vivo* evidence supporting their role in human epilepsy (LaCoursiere et al., 2024). More recently, CRISPR/Cas9-generated zebrafish mutants have been established for multiple epilepsy-related genes, including *napb* (Shin et al., 2025), *phf21ab* (Wang D. et al., 2025), *slc13a5* (Dogra et al., 2025), and *pnkp* (Wang G. et al., 2025), collectively demonstrating the utility of zebrafish for rapid *in vivo* functional validation of candidate epilepsy genes and for screening potential therapeutic interventions. Regarding physiological parameters, most epilepsy studies use larvae at 5–7 days post-fertilization (dpf) due to their optical transparency and suitability for high-throughput screening. Gender is rarely specified in larval studies as sexual dimorphism has not yet developed; adult studies are encouraged to report sex explicitly (Silva et al., 2022).

### 2.5 Insomnia model

Insomnia, a prevalent sleep disorder, is closely associated with various neurological conditions (Brandt, 2021; Dyken et al., 2012). Zebrafish, a diurnal vertebrate, possesses a sleep-wake regulatory system that closely resembles that of mammals, making it a prime candidate for investigating insomnia mechanisms and conducting drug screening (Yokogawa et al., 2007; Zhdanova, 2011). The caffeine-induced insomnia model is most widely used. Zebrafish juveniles exposed to caffeine concentrations ranging from 100 to 300 μmol/L exhibited reduced total nighttime sleep duration, heightened sleep fragmentation, increased frequency of awakenings, and prolonged sleep latency (Wei et al., 2024). These observed behavioral alterations align with adenosine receptor antagonism mechanisms, indicating a disruption in the equilibrium between sleep stress and wake-promoting networks (Reichert et al., 2022). In addition to caffeine, alternative chemical insomnia models have been developed. PTZ exposure via immersion triggers hyperactive locomotion and disrupted sleep architecture, providing a dependable model for screening sedative-hypnotic compounds and TCM interventions (Zhang X. et al., 2025). Similarly, para-chlorophenylalanine (PCPA) treatment has been utilized to induce insomnia by inhibiting serotonin synthesis, offering an additional validated approach (Li W. et al., 2024).

Circadian rhythm disruption represents another well-established insomnia model. Continuous exposure to light (24 h light: 0 h dark) or photoperiod inversion can disrupt circadian rhythms, leading to extended periods of activity and phase shifts that reduce intraday stability (Park et al., 2024). Prolonged infrared activity monitoring can assess these disturbances and detect changes in sleep patterns (Silva et al., 2022; Wang J. et al., 2023). Light exposure influences the expression of clock genes, indicating its impact on sleep and cognitive function mediated by rhythmic networks (Vatine et al., 2009). Artificial light at night (ALAN) of various wavelengths has also been shown to disrupt circadian rhythms and sleep behavior in zebrafish, with some spectral compositions exerting transgenerational effects (Li Y. et al., 2024).

The advent of gene editing technologies, particularly CRISPR/Cas9, has enabled the rapid generation of zebrafish lines carrying mutations in sleep-related genes. Hypocretin/orexin (Hcrt) overexpression induces an insomnia-like phenotype characterized by hyperarousal and dramatically reduced ability to initiate and maintain nighttime rest, directly recapitulating human insomnia (Prober et al., 2006). Conversely, disruption of Hcrt signaling results in sleep fragmentation mimicking narcolepsy. The hypocretin system in zebrafish is represented by a single *hcrt* gene and one Hcrt receptor (HcrtR), which is structurally closest to the mammalian HcrtR2, facilitating the study of evolutionarily conserved sleep circuits (Dyachuk, 2024). Additional transgenic models targeting sleep-regulatory genes, including HCN channel mutants and QRFP/Pth4 neuron-specific lines, have been established for high-throughput drug screening and mechanistic studies (Chiu et al., 2016; Doldur-Balli et al., 2024a). For insomnia studies, larvae at 5–7 dpf are most commonly used for chemical and light-based assays, whereas adult zebrafish (3–6 months) are employed when chronic effects or more complex sleep architecture is assessed. Gender differences have not been systematically reported, but researchers are advised to consider both sexes in adult studies (Doldur-Balli et al., 2024b).

### 2.6 Other neurological disease models

Beyond the aforementioned models, zebrafish have proven effective in modeling a wide spectrum of additional neurological and psychiatric disorders. For depression, zebrafish exhibit quantifiable behavioral phenotypes—including reduced locomotion, social withdrawal, and anhedonia—and diverse modeling approaches (genetic and pharmacological manipulations, chronic stress paradigms, and gut-brain axis studies) have been established, enabling investigations into the physiological, genetic, pharmacological, and environmental drivers of this disorder (Zhang et al., 2021; Zhang et al., 2018; Yang B. et al., 2025). For spinal cord injury (SCI), the remarkable regenerative capacity of the zebrafish central nervous system has been extensively leveraged; larval zebrafish, in particular, offer optical transparency for real-time visualization of cellular repair processes, and recent studies have systematically optimized injury protocols by comparing different developmental stages to standardize regeneration assays (Zeng and Tsai, 2023; Walker et al., 2025). For Huntington’s disease (HD), the expression of mutant HTT proteins recapitulates key pathological features including progressive motor dysfunction and neurodegeneration (Schiffer et al., 2007); more recently, a quinolinic acid-induced HD-like model in adult zebrafish has been validated, which recapitulates neurobehavioral impairments, oxidative stress, and neuroinflammation while enabling rapid screening of neuroprotective compounds targeting GSK-3β signaling (Goel et al., 2025). For amyotrophic lateral sclerosis (ALS), expression of mutant SOD1 or TDP-43 in zebrafish recapitulates hallmark pathologies including motor neuron degeneration, axonopathy, and locomotor deficits (Laird et al., 2010; Ramesh et al., 2010); recent work has further revealed that large spinal motor neurons, which are most susceptible to ALS, exhibit accelerated autophagic and proteasomal degradation that is amplified by TDP-43 loss, implicating catabolic stress as a potential therapeutic target (Asakawa et al., 2025). For autism spectrum disorders (ASDs) and other neurodevelopmental disorders, both genetic (e.g., *Shank3, Cntnap2, Neuroligin3, Arid1b* mutants) and environmental (e.g., valproate-induced) zebrafish models have been extensively validated, enabling *in vivo* dissection of synaptic dysfunction, excitatory/inhibitory imbalance, and social behavioral abnormalities (Meshalkina et al., 2018; Pal et al., 2025; Camussi et al., 2025). Collectively, these models have significantly enhanced our understanding of the pathogenesis of these complex diseases and continue to offer novel targets and approaches for therapeutic intervention (Meng et al., 2025).

## 3 Neuroprotective effects of TCM in zebrafish models

The zebrafish model, characterized by its holistic approach, visualization capabilities, and high-throughput features, serves as an ideal platform for systematically assessing the neuroprotective effects of TCM, which is a complex system. In recent years, numerous studies have employed the zebrafish model to elucidate the efficacy and mechanisms of TCM in the prevention and treatment of neurological disorders. These investigations have explored various levels, including individual botanical drugs, classical compound formulas, and active metabolites (Chakraborty et al., 2025; Dongjie et al., 2022; Lu et al., 2024).

### 3.1 Applications in Alzheimer’s disease research

Active metabolites from TCM have demonstrated neuroprotective effects in zebrafish AD models. Acteoside (ACT) from *Cistanche tubulosa* (Schenk) Wight [Orobanchaceae; Cistanches herba] alleviates AD-associated neuroinflammation by promoting microglial M1-to-M2 polarization via NF-κB and AMPK pathways (Li et al., 2021). Processed American ginseng saponins, particularly AGTS5 from *Panax quinquefolius* L. [Araliaceae; Panacis quinquefolii radix], reverse neuronal damage and cognitive deficits in AlCl_3_-induced zebrafish models through nerve repair, neurotransmitter regulation, and oxidative stress reduction (Yang Y. et al., 2025). Berberine chloride (BC) improves survival, reduces oxidative stress and neuroinflammation, and lowers lipid levels in AlCl_3_-exposed larvae, with molecular docking suggesting ABCA1 agonism (Uvarajan et al., 2025a). *Eucommia ulmoides* Oliv. [Eucommiaceae; Eucommiae cortex] olive male flower extract (EUMF) attenuates motor deficits, Aβ deposition, and neuronal apoptosis via autophagy regulation and acetylcholinesterase (AChE) inhibition (Sun et al., 2022). Arbutin restores antioxidant activity and alleviates cognitive dysfunction and neuroinflammation in AlCl_3_-exposed zebrafish (Uvarajan et al., 2025b). *Hericium erinaceus* (Bull.) Pers. [Hericiaceae] extract (a medicinal fungus, not a botanical drug) reduces cerebral oxidative stress and scopolamine-induced memory deficits (Valu et al., 2021). *Atractylodes macrocephala* Koidz. [Asteraceae; Atractylodis macrocephalae rhizoma] (AMK) ameliorates AD-like symptoms by modulating ferroptosis-related genes (*EGFR*, *HMOX1*) and improves behavioral responses and reduces neuronal apoptosis (Singh et al., 2024; Zheng et al., 2025). Linarin, a flavonoid from *Chrysanthemum indicum* L. [Asteraceae; Chrysanthemi indici flos], targets AChE and shows superior dyskinesia recovery compared to donepezil in zebrafish AD models (Pan et al., 2019).

Chinese botanical drug formulas and combination therapies also exhibit pleiotropic benefits. Shenghui Decoction (SHD; composed of *Panax ginseng* C.A.Mey., *Astragalus mongholicus* Bunge, *Gastrodia elata* Blume, among others) inhibits the JNK/p38 MAPK pathway, alleviating motor and cognitive deficits, oxidative stress, and neuroinflammation in AlCl_3_-induced zebrafish (Lu et al., 2024). In a comorbid Alzheimer’s disease and osteoporosis (AD-OP) zebrafish model, the combination of osthole (OST) and notopterol (NOT) – metabolites from *Angelica pubescens* Maxim. [Apiaceae; Angelicae pubescentis radix] and *Notopterygium incisum* K.C.Ting ex H.T.Chang [Apiaceae; Notopterygii rhizoma et radix], respectively – exerts synergistic anti-inflammatory effects, suppressing NO and ROS release more effectively than either compound alone (Guo et al., 2024). Similarly, *Cistanche tubulosa* (Schenk) Wight [Orobanchaceae; Cistanches herba] has been identified as a dual-target therapeutic for AD-OP comorbidity (Li C. et al., 2020).

Nanotechnology-based delivery of TCM metabolites has been validated in zebrafish models. Curcumin-loaded liposomes (curcumin from *Curcuma longa* L. [Zingiberaceae; Curcumae longae rhizoma]) penetrate the brain and reduce oxidative stress in neurons, enabling targeted neuroprotection (Fernandes et al., 2021). Cinnamic acid hybrids act as multi-target agents (huBuChE, MAO-B), cross the blood-brain barrier, and improve cognitive impairment without acute toxicity (Wang L. et al., 2021). Gelatin/PLA/gold nanocomposites (Ge/PLA/AuNCs) synthesized with *Syzygium cumini* (L.) Skeels [Myrtaceae] extract exhibit good biocompatibility and inhibit AChE and butyrylcholinesterase (BChE), demonstrating anti-AD effects (Rajkumar et al., 2025). Collectively, these studies confirm the efficacy of TCM-derived metabolites, formulas, and nano-formulations in improving AD-related functional impairments in zebrafish models. A summary of representative studies is presented in Table 1.

**TABLE 1:** Neuroprotective effects and mechanisms of TCM-Derived metabolites and nanotechnology-based strategies in zebrafish models of AD.

Category	Metabolite/Strategy	Source/Characteristics	Model Inducer	Neuroprotective Effects	Mechanism of Action	References
TCM active metabolites	Acteoside (ACT)	*Cistanche tubulosa* (Schenk) Wight [Orobanchaceae; Cistanches herba]	AlCl_3_	Alleviates AD-associated neuroinflammation	Regulates NF-κB/AMPK pathways; promotes M1→M2 microglial transformation	Li et al. (2021)
	Ginsenoside AGTS5	Rare ginsenoside from steamed *Panax quinquefolius*L. [Araliaceae; Panacis quinquefolii radix]	AlCl_3_	Reverses neuronal damage; facilitates nerve repair/regeneration; improves cognitive impairment	Repairs damaged nerve cells; regulates neurotransmitters; alleviates oxidative stress/inflammation	Yang B. et al. (2025)
	Berberine chloride (BC)	Alkaloid from *Coptis chinensis* Franch. [Ranunculaceae; Coptidis rhizoma] and other plants	AlCl_3_	Alleviates oxidative stress/neuroinflammation; improves survival; reduces lipid levels	Potential ABCA1 agonist (molecular docking prediction)	Uvarajan et al. (2025a)
	*Eucommia ulmoides* olive male flower extract (EUMF)	*Eucommia ulmoides* Oliv. [Eucommiaceae; Eucommiae cortex]	AlCl_3_	Mitigates motor disorders; inhibits aβ deposition; reduces apoptosis	Regulates autophagy, AChE activity, and dopamine transporter activity	Sun et al. (2022)
	Arbutin	Natural metabolite from *Arctostaphylos uva-ursi* (L.) Spreng. [Ericaceae] and other plants	AlCl_3_	Improves cognitive function; reduces oxidative stress; alleviates neuroinflammation	Reduces ROS levels; restores antioxidant activity	Uvarajan et al. (2025b)
	*Hericium erinaceus* ethanolic extract	Medicinal fungus *Hericium erinaceus* (Bull.) Pers. [Hericiaceae] (not a botanical drug)	Scopolamine	Reduces cerebral oxidative stress; ameliorates memory disorders	Decreases oxidative stress and AChE activity	Valu et al. (2021)
	*Atractylodes Macrocephala Koidz*. (AMK)	*Atractylodes macrocephala*Koidz. [Asteraceae; atractylodis macrocephalae rhizoma]	Scopolamine	Alleviates AD-like symptoms; improves behavioral responses; reduces neuronal apoptosis	Modulates ferroptosis-related genes (EGFR, HMOX1); regulates oxidative phosphorylation/lysosomal pathways	Singh et al., 2024; Zheng et al., 2025
	Linarin	Flavonoid glycoside from *Chrysanthemum indicum* L. [asteraceae; Chrysanthemi indici flos]	AlCl_3_	Improves dyskinesia recovery; inhibits AChE activity	Targets AChE active sites (molecular docking confirmed); inhibits AChE activity	Pan et al. (2019)
TCM botanical drug combinations	Shenghui decoction (SHD)	Ancient clinical TCM formula (composition: *Panax ginseng*C.A.Mey., *Astragalus mongholicus* Bunge, *Gastrodia elata* Blume, etc.)	AlCl_3_	Improves motor/cognitive deficits, anxiety, and aggression; alleviates oxidative stress/inflammation	Inhibits JNK/p38 MAPK pathway	Lu et al. (2024)
​	Osthole (OST) + notopterol (NOT)	Combination of metabolites from *Angelica pubescens*Maxim. [Apiaceae; angelicae pubescentis radix] and *Notopterygium incisum*K.C.Ting ex H.T.Chang [apiaceae; Notopterygii rhizoma et radix]	AD-OP comorbid model (AlCl_3_, Prednisolone)	Exerts synergistic anti-inflammatory effects; inhibits NO/ROS release	Synergistic regulation of inflammatory responses	Guo et al., 2024; Li Y. Q. et al., 2020
Nanotechnology-based strategies	Curcumin-loaded liposomes	Liposomal carrier loaded with curcumin (from *Curcuma longa* L. [Zingiberaceae; Curcumae longae rhizoma])	Not specified	Reduces oxidative stress in SH-SY5Y neurons; targeted neuroprotection	Biocompatible; penetrates embryos; accumulates in brain/yolk sac (lipid-rich regions)	Fernandes et al. (2021)
	Cinnamic acid hybrids	Cinnamic acid-derived hybrid metabolites	AlCl_3_	Enhances motor disorder recovery and response efficiency; improves memory impairment	Displays favorable blood-brain barrier penetration; inhibits huBuChE and MAO-B activity; exhibits anti-inflammatory and neuroprotective effects	Wang L. et al. (2021)
	Ge/PLA/AuNCs	Gelatin/PLA/gold nanocomposites with *Syzygium cumini* (L.) Skeels [Myrtaceae] extract	Not specified	Exerts anti-AD effects; biocompatible and safe	Inhibits AChE and BChE activity	Rajkumar et al. (2025)

### 3.2 Applications in Parkinson’s disease research

Active metabolites from TCM exhibit neuroprotective effects in zebrafish PD models. Panaxatriol saponins (PTS) from *Panax notoginseng* (Burkill) F.H.Chen [Araliaceae; Notoginseng radix et rhizoma] reduce 6-OHDA-induced dopaminergic neuron loss and improve motor behavior (Zhang et al., 2017a). Schisantherin A (SA) from *Schisandra chinensis* (Turcz.) Baill. [Schisandraceae; Schisandrae chinensis fructus] alleviates oxidative stress and neuronal injury via iNOS suppression and MAPK/PI3K/Akt/GSK3β pathways (Zhang et al., 2015). *Calendula officinalis*L. [Asteraceae; Calendulae flos] extract (ECoL) activates autophagy, upregulates autophagy-related genes, and promotes α-synuclein clearance, ameliorating MPTP-induced motor deficits (Wang M. et al., 2023). *Polygala tenuifolia* Willd. [Polygalaceae; Polygalae radix] extracts (PRE) downregulate *pck1* to regulate glucose metabolism, alleviating both PD and depressive symptoms (Jiao et al., 2026). Berberine (BBR) from *Coptis chinensis* Franch. [Ranunculaceae; Coptidis rhizoma] and other plants exerts hormetic neuroprotection via PI3K/AKT/Bcl-2 and Nrf2/HO-1 pathways (Zhang et al., 2017a). Its fluorescently labeled derivative BBRP crosses the blood-brain barrier, targets mitochondria, and shows anti-PD efficacy (Wang X. et al., 2021).

Natural metabolites also demonstrate anti-PD potential. Mangiferin (MGF) from *Mangifera indica* L. [Anacardiaceae] maintains mitochondrial homeostasis by regulating PD-related genes (*lrrk2*, *vps35*, *atp13a*, *dnajc6*, *uchl1*) and restores SOD/CAT activities (Qin et al., 2024). Hesperidin from citrus fruits downregulates *lrrk2*, *gsk3β*, *casp3*, *casp9*, and *polg*; naringenin reduces oxidative stress and protects mitochondrial membrane potential; both improve motor patterns (Kesh et al., 2021a; 2021b). Chlorogenic acid (CGA) from coffee and other plants alleviates MPTP-induced symptoms by promoting autophagy via *α-syn* and *lc3b* regulation (Gao et al., 2023). Acteoside activates Nrf2-ARE signaling (Li et al., 2018). Theacrine from Chinese tea *Kucha* (*Camellia assamica* var. *kucha*) reduces ROS and enhances mitochondrial function via SIRT3-mediated SOD2 deacetylation (Duan et al., 2020).

TCM compound formulas show synergistic effects. Tongtian Oral Liquid (TTKFY; composition includes *Panax ginseng* C.A.Mey., *Astragalus mongholicus* Bunge, etc.) protects dopaminergic neurons, improves behavior, and regulates dopamine pathway mRNAs in a dose-dependent manner (Dongjie et al., 2022). Qilong Capsule (QLC; derived from Buyang Huanwu Decoction, containing *Astragalus mongholicus*, *Panax notoginseng*, *Salvia miltiorrhiza*, among others) reduces dopaminergic neuron loss and apoptosis by inhibiting mitochondrial apoptosis, promoting autophagy, and degrading α-synuclein (Mo et al., 2023).

Fungal metabolites and nanotechnology-based delivery have been validated. Granulathiazole A (from fungal sources) inhibits ferroptosis via SLC7A11/GPX4 and reduces α-syn aggregation (Kong et al., 2024). Brassisterol A mitigates neuroinflammation through NLRP3/caspase-1/GSDMD signaling (Tong et al., 2024). *Sanghuangporus vaninii* (Ljub.) L.W. Zhou and Y.C. Dai [Hymenochaetaceae] mycelium extracts (SvMEs) reverse MPTP-induced dopaminergic loss and movement disorders (Li et al., 2022). Nanocarriers improve delivery: ginkgolide B nanocrystals (GB-NCs) and PEG-PCL nanoparticles enhance brain accumulation, protect neurons, and restore dopamine levels (Liu et al., 2020; Zhao et al., 2020). Schisantherin A nanocrystals (SA-NCs) activate the Akt/Gsk3β pathway (Chen T. et al., 2016). Puerarin nanocrystals (PU-NCs) and FRET-confirmed nanoparticles improve oral absorption and brain penetration, ameliorating MPTP-induced deficits (Xiong et al., 2019; Chen et al., 2019). Collectively, these studies confirm the efficacy of TCM -derived metabolites, compound formulas, fungal metabolites, and nano-formulations in improving PD-related functional impairments in zebrafish models, providing a solid foundation for novel anti-PD strategies and clinical translation. An overview of key findings is provided in Table 2.

**TABLE 2:** Neuroprotective effects and mechanisms of TCM-Derived metabolites and related strategies in zebrafish models of PD.

Category	Metabolite/Strategy	Source/Characteristics	Model Inducer	Neuroprotective Effects	Mechanism of Action	References
TCM active metabolites	Panaxatriol saponins (PTS)	*Panax notoginseng (Burkill) F.H.Chen [Araliaceae; Notoginseng radix et rhizoma]*	6-OHDA	Mitigates dopaminergic neuron loss; improves motor behavior	Upregulates PI3K/AKT/mTOR and AMPK/SIRT1/FOXO3 pathways	Zhang et al. (2017a)
	Schisantherin a (SA)	*Schisandra chinensis* (Turcz.) Baill. [Schisandraceae; Schisandrae chinensis fructus]	6-OHDA	Alleviates oxidative stress and neuronal injury	Inhibits iNOS expression; regulates MAPK, PI3K/Akt, GSK3β pathways	Zhang et al. (2015)
	Extract of Calendula officinalis L. (ECoL)	*Calendula officinalis* L. [asteraceae; Calendulae flos]	MPTP	Reduces dopaminergic neuron loss and neurovascular impairment; suppresses movement disorders	Activates autophagic pathway; upregulates autophagy-related genes; promotes α-synuclein aggregation and degradation	Wang M. et al. (2023)
	Polygalae radix extracts (PRE)	*Polygala tenuifolia* Willd. [Polygalaceae; Polygalae radix]	MPTP	Alleviates PD and depression-related symptoms	Downregulates *pck1* expression; regulates glucose metabolism-related pathways	Jiao et al. (2026)
	Berberine (BBR)	From numerous medicinal plants (e.g., *Coptis chinensis*)	6-OHDA	Mitigates dopaminergic neuron loss and behavioral deficits	Upregulates PI3K/AKT/Bcl-2 and Nrf2/HO-1 signaling pathways	Zhang et al. (2017b)
	Fluorescently labeled berberine derivative (BBRP)	Berberine derivative	MPTP	Exerts neuroprotective effects against PD-like symptoms; reduces dopaminergic neuron loss	Crosses blood-brain barrier; localizes in mitochondria; protects mitochondria	Wang X. et al. (2021)
Natural metabolites	Mangiferin (MGF)	From mango (*Mangifera indica* L.) fruits, leaves, and bark	MPTP	Maintains mitochondrial homeostasis; restores antioxidant enzyme activities	Regulates *LRRK2, VPS35* gene expression; restores SOD, CAT activities	Qin et al. (2024)
	Hesperidin (HES)	From citrus fruits (grapefruit, oranges, lemon, tangerine)	6-OHDA	Enhances motor patterns	Downregulates *LRRK2, GSK3β, casp3, casp9, polg* genes	Kesh et al. (2021a)
	Naringenin	From citrus fruits, tomatoes, figs	6-OHDA	Reduces oxidative stress; enhances motor patterns	Reduces oxidative stress markers; protects mitochondrial membrane potential	Kesh et al. (2021b)
Natural metabolites	Chlorogenic acid (CGA)	From coffee, honeysuckle, eucommia	MPTP	Alleviates PD-like symptoms	Regulates *α-syn, lc3b* gene expression; promotes autophagy	Gao et al. (2023)
	Acteoside	From many dicotyledonous plants	6-OHDA	Protects dopaminergic neurons against damage	Activates Nrf2-ARE signaling pathway	Li et al. (2018)
	Theacrine	From Chinese tea *Kucha* (*Camellia assamica* var. *kucha*)	MPTP	Enhances mitochondrial function	Reduces ROS accumulation; SIRT3-mediated deacetylation of SOD2	Duan et al. (2020)
TCM prescriptions	Tongtian oral liquid (TTKFY)	TCM prescription	MPTP	Protects dopaminergic neurons; improves behavioral deficits; enhances antioxidant activity	Regulates expression of dopamine pathway-related mRNAs	Dongjie et al. (2022)
	Qilong capsule (QLC)	Derived from Buyang Huanwu decoction	MPTP	Mitigates dopaminergic neuron damage; reduces apoptotic cells; alleviates dyskinesia	Inhibits mitochondrial apoptosis; promotes autophagy; degrades α-synuclein; fosters neuronal growth; rescues impaired neuronal function	Mo et al. (2023)
Fungal metabolites	Granulathiazole A	Fungal metabolite	6-OHDA	Alleviates locomotor aberration; reduces α-synuclein aggregation	Activates Nrf2/HO-1 pathway; inhibits ferroptosis	Kong et al. (2024)
	Brassisterol A	Fungal metabolite	MPTP	Ameliorates locomotor aberration; protects dopaminergic cells	Modulates NLRP3/caspase-1/GSDMD pathway; mitigates neuroinflammation	Tong et al. (2024)
	*Sanghuangprous vaninii*mycelium extracts (SvMEs)	*Sanghuangporus vaninii* (Ljub.) L.W. Zhou and Y.C. Dai [Hymenochaetaceae]	MPTP	Reverses dopaminergic neuron loss and neurovascular reduction; alleviates movement disorders	Alleviates oxidant stress and accelerates α-synuclein degradation	Li et al. (2022)
Nanotechnology-based delivery systems	Ginkgolide B nanocrystals (GB-NCs)	Ginkgolide B-loaded nanocrystals; rapid dissolution, high oral bioavailability, excellent brain uptake	MPP^+^	Protects neurons against cytotoxicity; improves behavioral performance; alleviates dopamine deficiency; increases dopamine metabolite levels	Nanoparticle characteristics facilitate efficacy	Liu et al. (2020)
	PEG-PCL nanoparticles (ginkgolide B carrier)	PEG-PCL nanoparticles; enables sustained drug release, enhances brain accumulation	Not specified (validated in zebrafish models)	Enhances anti-PD efficacy	Enables sustained drug release; enhances brain accumulation; crosses physiological barriers	Zhao et al. (2020)
	Schisantherin a nanocrystals (SA-NC)	Schisantherin A-loaded nanocrystals; improves bioavailability and intracranial concentration of BCS class II compounds	MPTP	Reverses DA neuronal loss and locomotion deficiency; exerts neuroprotective effects	Activates Akt/Gsk3β pathway	Chen T. et al. (2016)
	Puerarin-loaded nanoparticles (PU-NCs)	Puerarin-loaded nanoparticles; orally absorbable, rapid brain penetration	MPTP	Attenuates dopamine depletion; ameliorates behavioral deficits; increases dopamine and metabolite levels	Nanoparticle characteristics facilitate targeted delivery	Chen et al., 2019; Xiong et al., 2019

### 3.3 Applications in cerebral ischemia studies

Active metabolites from TCM exhibit vascular protective, antithrombotic, and neuroprotective effects in zebrafish models of ischemic stroke. Ginsenoside F1 (GF1) from *Panax ginseng* C.A.Mey. [Araliaceae; Ginseng radix et rhizoma] alleviates axitinib-induced vascular damage and enhances angiogenesis via the IGF-1/IGF1R pathway, improving recovery from ischemic stroke (Zhang J. et al., 2019). *Caragana jubata* (Pall.) Poir. [Fabaceae] extract (ECJ; Tibetan medicine, not officially listed) reduces cerebral thrombosis incidence in a dose-dependent manner, with efficacy superior to aspirin (Zhao et al., 2023). The combination of *Centella asiatica* (L.) Urb. [Apiaceae; Centellae herba] hydroalcoholic extract (HA-CA) with intermittent fasting mitigates behavioral deficits, oxidative stress, neuroinflammation, and mitochondrial dysfunction induced by subacute hypoxia (Bindal et al., 2024). Ilexsaponin A1 from *Ilex pubescens* Hook. and Arn. [Aquifoliaceae; Ilicis pubescentis radix] shows pro-angiogenic activity in zebrafish (Li J. et al., 2017). *Rubia cordifolia* L. [Rubiaceae; Rubiae radix et rhizoma] extract (QC) exerts dual antithrombotic and pro-angiogenic effects (Chen et al., 2018). *Polygoni Cuspidati* rhizoma et radix extract (PCRR) from *Reynoutria japonica* Houtt. (syn. *Polygonum cuspidatum*) [Polygonaceae; Polygoni cuspidati rhizoma et radix] inhibits VEGF-induced angiogenesis via VEGFR2 signaling (Hu et al., 2018). Protoparaxotriol saponins (PTS) from *Panax notoginseng* (Burkill) F.H.Chen [Araliaceae; Notoginseng radix et rhizoma] alleviate thrombosis by accelerating blood flow and regulating coagulation-, inflammation-, and apoptosis-related genes, including inhibition of NF-κB and PI3K/AKT pathways (Liu et al., 2024). Muscone and l-borneol (natural products) targeting TRPV1 and TRPM8 exert synergistic neuroprotective effects in zebrafish antithrombotic and anti-ischemic models, regulating Ca^2+^ concentration and energy metabolism (Ma L. et al., 2025).

TCM compound formulas also show therapeutic benefits. *Salvia miltiorrhiza* Bunge [Lamiaceae; Salviae miltiorrhizae radix et rhizoma] and *Panax notoginseng* combined at a 10:1 ratio achieve optimal antithrombotic efficacy, downregulating thrombosis-related genes (Yin et al., 2020). Angong Niuhuang Pill (ANP; a classic TCM formula containing *Bovis calculus*, *Moschus*, etc.) and its combinatorial metabolite BECCs V reduce motor impairments and neuronal damage in zebrafish cerebral ischemia models via PI3K/AKT signaling (Zhang C. et al., 2025). Sanwujiao Granule (SW) exerts angiogenic effects in early administration, alleviating ischemic stroke injury (Zhou et al., 2024). Qilong Capsule (QLC; derived from Buyang Huanwu Decoction) alleviates ponatinib-induced ischemic stroke by regulating coagulation, inflammation, and apoptosis (Lin et al., 2023). Shuxinyin Formula promotes angiogenesis via VEGF/PI3K/Akt/MAPK signaling (Zhou et al., 2019). Dang-Gui-Si-Ni (DGSN) Decoction exerts anticoagulant activity by regulating multiple coagulation factors (Li W. et al., 2024).

TCM injections have been validated in zebrafish models. Guhong Injection (GHI) reduces NF-κB-mediated pro-inflammatory cytokines and exhibits antithrombotic effects by decreasing coagulation factors and inflammatory cytokines (Wang Y. et al., 2022). This research introduces a novel approach to drug discovery through multi-phenotypic screening, offering a new perspective for identifying active metabolites and elucidating the mechanisms of TCM injections. Table 3 summarizes the effects and mechanisms of TCM metabolites, formulas, and injections in zebrafish models of ischemic stroke and thrombosis.

**TABLE 3:** Effects and mechanisms of TCM active metabolites, prescriptions and injections in zebrafish models of ischemic stroke and thrombosis.

Category	Metabolite/Formula/Injection	Source/Characteristics	Model Type (Zebrafish)	Core Effects	Mechanism of Action	References
TCM active metabolites	Ginsenoside F1 (GF1)	Active metabolite from *Panax ginseng* C.A.Mey. [araliaceae; ginseng radix et rhizoma]	Axitinib-induced vascular injury; ischemic stroke	Alleviates vascular damage; promotes angiogenesis; improves cerebrovascular function	Activates IGF-1/IGF1R pathway	Zhang J. et al. (2019)
	*Caragana jubata* ethanol extract (ECJ)	95% ethanol extract of *Caragana jubata* (Pall.) Poir. [Fabaceae] (Tibetan medicine, not officially listed)	Ponatinib-induced cerebral thrombosis	Reduces thrombosis incidence in a dose-dependent manner; superior to aspirin	Enhances cell viability; modulates microglial polarization	Zhao et al. (2023)
	*Centella asiatica*hydroalcoholic extract (HA-CA) + intermittent fasting (IF)	Combination of *Centella asiatica* (L.) Urb. [Apiaceae; Centellae herba] extract with intermittent fasting	Subacute hypoxia model	Improves behavioral abnormalities, oxidative stress, neuroinflammation and mitochondrial dysfunction	Improves abnormal hypoxia-related signaling molecules	Bindal et al. (2024)
	Ilexsaponin A1	Saponin metabolite from *Ilex pubescens* Hook. and arn. [Aquifoliaceae; Ilicis pubescentis radix]	VEGF VRI-induced vascular insufficient model	Exerts pro-angiogenic activity *in vitro* and *in vivo*	Activates Akt/mTOR, MAPK/ERK and Src- and FAK-dependent signalling pathways	Li Y. et al. (2017)
	*Rubia cordifolia* extract (QC)	Extract of *Rubia cordifolia* L. [Rubiaceae; Rubiae radix et rhizoma] containing 12 key metabolites	PHZ-induced thrombosis	Antithrombotic; pro-angiogenic	Exerts dual effects through 12 key compounds	Chen et al. (2018)
	*Polygoni Cuspidati* rhizoma et radix extract (PCRR)	Extract of root and rhizome of *Reynoutria japonica* Houtt. (syn. *Polygonum cuspidatum*) [Polygonaceae; Polygoni cuspidati rhizoma et radix]	Incubated with avastin	Inhibits abnormal angiogenesis	Regulates the VEGFR2 signaling pathway	Hu et al. (2018)
	Protoparaxotriol saponins (PTS)	Saponin metabolites from *Panax notoginseng*	Ponatinib-induced thrombosis	Alleviates thrombosis; accelerates blood flow	Regulates coagulation, inflammation and apoptosis-related genes; inhibits NF-κB and PI3K/AKT pathways	Liu et al. (2024)
TCM active metabolites	Muscone + l-borneol	Natural drug combination (multitargeted candidates)	Melanin allele mutant zebrafish (albino) given arachidonic acid	Synergistically exerts anti-ischemic and antithrombotic effects	Regulates TRPV1/TRPM8; modulates Ca^2+^ concentration and energy metabolism via purine/amino acid metabolism	Ma L. et al. (2025)
TCM prescriptions/Classic formulas	*Salvia miltiorrhiza* - *Panax notoginseng* (10:1)	TCM compatibility combination	PHZ-induced thrombosis	Optimal antithrombotic efficacy	Downregulates the expression of thrombosis-related genes	Yin et al. (2020)
	Angong Niuhuang pill (ANP) and BECCs V	Classic TCM formula and its combinatorial active metabolite	Ponatinib-induced model	Neuroprotective; improves motor impairment; alleviates neuronal damage	Regulates the PI3K/AKT signaling pathway	Zhang C. et al. (2025)
	Sanwujiao granule (SW)	TCM granule	Vascular deficiency model of Tg (kdrl:eGFP)	Promotes angiogenesis	Affects angiogenesis-associated targets	Zhou et al. (2024)
	Qilong capsule (QLC)	TCM capsule	Ponatinib-induced ischemic stroke	Alleviates ischemic stroke symptoms	Regulates coagulation, inflammation and apoptosis pathways	Lin et al. (2023)
	Shuxinyin formula	TCM prescription	Transgenic zebrafish line Tg (fli1a: EGFP) treated VRI	Significant pro-angiogenic effect	Regulates VEGF/PI3K/Akt/MAPK signaling pathway	Zhou et al. (2019)
	Dang-Gui-Si-Ni (DGSN) decoction	Classic TCM formula	Ponatinib-induced thrombosis	Anticoagulant activity	Simultaneously regulates multiple coagulation factors	Li W. et al. (2024)
TCM injections	Guhong injection (GHI)	Clinically commonly used TCM injection	PHZ-induced thrombosis and ponatinib-induced cerebral ischemia	Antithrombotic; anti-inflammatory	Reduces NF-κB-mediated pro-inflammatory factor expression; decreases release of coagulation and inflammatory factors	Wang Q. et al. (2022)

### 3.4 Applications in epilepsy research

Active metabolites from TCM exhibit antiepileptic activity in zebrafish models. Parishin E and N^6^-p-hydroxybenzyladenosine from *Gastrodia elata* Blume [Orchidaceae; Gastrodiae rhizoma] regulate neurotransmitter pathways (Chen S. et al., 2024). Steroidal saponins from *Solanum torvum*Sw. [Solanaceae] (Water Eggplant, not officially listed) show moderate antiepileptic activity in PTZ-induced seizures (Ren et al., 2024). Schaftoside attenuates seizures by inhibiting apoptosis, inflammation, and oxidative stress (Dang et al., 2021). *Arisaema heterophyllum* Blume [Araceae; Arisaematis rhizoma] extract prolongs seizure onset latency and reduces seizure-like behaviors via neuroinflammation regulation, targeting Anxa1c, Il1b, and Ptger1a (Gao et al., 2025).

Among monomeric metabolites, berberine and palmatine have been extensively studied. Berberine (from *Coptis chinensis* Franch. [Ranunculaceae; Coptidis rhizoma] and other plants) pretreatment improves memory consolidation and delays seizure progression in PTZ-induced cognitive impairment, whereas hesperidin shows no significant efficacy (Bertoncello et al., 2024). Berberine and its derivatives, particularly BBR-D1, prolong seizure latency and suppress behaviors via anti-inflammatory pathways (Zhang et al., 2020). Palmatine (from *Berberis sibirica* Pall. [Berberidaceae]) exhibits anticonvulsant activity in zebrafish and mice, interacting with glutamic acid decarboxylase and AMPA receptors (Nieoczym et al., 2024). Palmatine reduces hyperlocomotion and alters neurochemical levels; its combination with berberine produces synergistic antiepileptic effects (Gawel et al., 2020).

Mechanistic studies identify potential targets. Reduced syntaxin 1B (STX1B) expression correlates with increased seizure activity; berberine upregulates STX1B, mediating its anticonvulsant effects (Zheng et al., 2018). *Magnolia officinalis* Rehder and E.H.Wilson [Magnoliaceae; Magnoliae officinalis cortex] extract and its metabolite magnolol show potent anticonvulsant effects in drug-resistant epilepsy models (Li Y. Q. et al., 2020). Tanshinone IIA from *Salvia miltiorrhiza* Bunge [Lamiaceae; Salviae miltiorrhizae radix et rhizoma] displays clear anticonvulsant activity in zebrafish seizure models (Buenafe et al., 2013).

Other natural products also show promise. Seven coumarin derivatives exhibit significant antiepileptic effects (Kozioł et al., 2021). The Kunitz-like peptide AdKuz2 (from the branching coral *Acropora digitifera* Dana, 1846) reduces glutamate synthesis and enhances GABA biosynthesis (Chen et al., 2022). Glycyrrhizin (GL) from *Glycyrrhiza* radix alleviates memory dysfunction in chronic seizures via the HMGB1-TLR4-NF-κB axis, acting as both an anticonvulsant and neuroprotective agent (Paudel et al., 2021).

TCM compound prescriptions demonstrate synergistic effects. Rongchang Capsule and Xifeng Capsule (classic TCM formulas) alleviate seizure-like behaviors and downregulate c-fos expression, likely via serotonin, GABA, and histamine pathways (Zhang S. et al., 2019). These studies highlight the potential of TCM-derived metabolites and other natural products as candidates for antiepileptic drug development, with zebrafish models serving as a robust platform for mechanistic studies and clinical translation. A detailed summary of antiepileptic effects and mechanisms is presented in Table 4.

**TABLE 4:** TCM-derived metabolites and natural products: Antiepileptic effects and mechanisms in zebrafish models.

Category	Metabolite/Extract	Source	Model Inducer	Antiepileptic Effects	Mechanism of Action	References
TCM active metabolites	Parishin E, N^6^-p-hydroxybenzyladenosine	*Gastrodia elata Blume [Orchidaceae; Gastrodiae rhizoma]*	PTZ	Exhibits antiepileptic activity; reduces number of whirls in zebrafish	Regulates neurotransmitter pathways	Chen S. et al. (2024)
	Steroidal saponins	*Solanum torvum* sw. [Solanaceae] (water eggplant, not officially listed)	PTZ	Exhibits antiepileptic activity; reduces locomotor activity	Not specified	Ren et al. (2024)
	Schaftoside	From *Rhizoma arisaematis*, *Eleusine indica*, *Glycyrrhiza uralensis*, *Dendrobium nobile*, *Lysimachia christinae*	PTZ	Suppresses seizure-like behavior; prolongs seizure onset	Inhibits apoptosis; downregulates inflammatory responses; alleviates oxidative stress	Dang et al. (2021)
	Ethanol extract of *Arisaema heterophyllum* Blume (BLU)	*Arisaema heterophyllum*Blume [araceae; arisaematis rhizoma]	PTZ	Prolongs seizure onset latency; reduces seizure-like behaviors; suppresses EEG activity	Regulates neuroinflammation (targets: Anxa1c, Il1b, Ptger1a)	Gao et al. (2025)
TCM-derived monomers	Berberine	*Berberis* spp., *Coptis* spp., and other plants	PTZ	Improves memory consolidation; delays seizure progression; prolongs seizure onset latency; suppresses seizure-like behaviors; alleviates hyperexcitatory movements	Regulates inflammatory responses; upregulates STX1B expression	Bertoncello et al., 2024; Zheng et al., 2018
	Berberine derivatives (BBR-D1)	Derivatives of berberine	PTZ	Prolongs seizure onset latency; suppresses seizure-like behaviors (BBR-D1 exhibits strongest effect)	Regulates inflammatory responses	Zhang et al. (2020)
	Palmatine	*Berberis sibirica* Pall. radix [Berberidaceae]	PTZ	Exhibits anticonvulsant activity in multiple models; reduces hyperlocomotion; alters neurochemical levels; shows dose-dependent antiepileptic activity	Interacts with glutamic acid decarboxylase and AMPA receptors; exerts synergistic effects with berberine	Nieoczym et al., 2024; Gawel et al., 2020
	Magnolol	*Magnolia officinalis* Rehder and E.H.Wilson [Magnoliaceae; Magnoliae officinalis cortex]	PTZ; ethylketopentenoate (EKP)	Shows potent anticonvulsant effects in drug-resistant epilepsy model	Not specified	Li C. et al. (2020)
TCM-derived monomers	Tanshinone IIA	*Salvia miltiorrhiza* Bunge [Lamiaceae; Salviae miltiorrhizae radix et rhizoma]	PTZ	Inhibits seizure activity (reduces PTZ-related activity)	Reduces *c-fos* expression	Buenafe et al. (2013)
Other natural products	Natural coumarin derivatives (7 types)	Natural products	PTZ	Decreases seizure-like behavior (PTZ-induced elevated locomotor activity); exhibits antiepileptiform activity in LFP recordings	Molecular simulations show interaction with GABA_A_ receptor	Kozioł et al. (2021)
	AdKuz2 (Kunitz-like peptide)	Branching coral *Acropora digitifera* (Dana, 1846)	PTZ	Shows remarkable antiepileptic efficacy	Reduces glutamate synthesis; enhances GABA biosynthesis	Chen et al. (2022)
	Glycyrrhizin (GL)	*Glycyrrhiza* radix (HMGB1 inhibitor)	PTZ	Exhibits anticonvulsant effects; alleviates memory dysfunction from chronic seizures	Regulates HMGB1-TLR4-NF-κB axis; exerts neuroprotective effects	Paudel et al. (2021)
TCM prescriptions	Rongchang capsule, Xifeng capsule	TCM prescriptions	PTZ	Alleviates seizure-like behaviors; downregulates c-fos expression	Alters signaling pathways of serotonin, GABA, and histamine	Zhang S. et al. (2019)

### 3.5 Applications in insomnia research

Zebrafish have emerged as a pivotal research model for investigating TCM-based insomnia treatments, attributed to their quantifiable sleep behaviors and high homology of neural pathways with humans. Zebrafish models, particularly the caffeine-induced insomnia model, have been widely used to evaluate TCM-based treatments through behavioral and molecular analyses (Wei et al., 2024; Doldur-Balli et al., 2024a).

Active metabolites from botanical drugs exhibit sleep-regulating properties. *Ziziphus jujuba* Mill. var. *spinosa* (Bunge) Hu ex H.F.Chow [Rhamnaceae; Ziziphi spinosae semen] (Semen Ziziphi Spinosae) reduces arousal and promotes resting states via the inhibitory neurotransmitter system (Xia et al., 2019). Ginsenoside Rg1 from *Panax ginseng* C.A.Mey. [Araliaceae; Ginseng radix et rhizoma] alleviates sleep deprivation-induced cognitive deficits and brain damage via the Bcl-2/Bax/caspase-3 pathway (Lu et al., 2023). Probiotic-fermented *Gastrodia elata* Blume [Orchidaceae; Gastrodiae rhizoma] outperforms unfermented material in maintaining serotonin balance through gut microbiota and amino acid metabolism (Zhang X. et al., 2025), and modulates neuroactive ligand-receptor interactions and DNA repair (Zhang et al., 2024). *Schisandra chinensis* (Turcz.) Baill. [Schisandraceae; Schisandrae chinensis fructus] extract shows neuroactive properties (Wang et al., 2018). *Ganoderma leucocontextum* (a medicinal fungus, not a botanical drug) extracts reduce locomotor activity via multi-component, multi-target mechanisms (Zhao et al., 2025).

TCM compound formulas demonstrate synergistic effects. Anmeidan (a TCM compound prescription) repairs circadian rhythms and regulates energy metabolism by downregulating clock genes, increasing ATPase activity, and modulating the OXA/p38 MAPK/ERK1/2 pathway (Wang Y. et al., 2022; Ye et al., 2024). Huanglian Wendan Decoction (HWD) exerts anti-insomnia effects by activating GABA_A_ receptors, with ethyl glucoside as a key metabolite (Li Y. et al., 2024). Chaihu Longgu Muli Decoction (CLMD) improves sleep-wake behaviors and reduces anxiety via IL6 and TNF pathways (Fan et al., 2025).

The zebrafish model provides a standardized, high-throughput evaluation system for investigating TCM treatments for insomnia, reinforcing the “multi-target synergy” concept of TCM and facilitating the modernization of TCM in sleep disorder management. Table 5 summarizes the efficacy and mechanisms of TCM interventions for insomnia evaluated in zebrafish models.

**TABLE 5:** TCM for insomnia: Efficacy and mechanisms evaluated in zebrafish models.

Category	Metabolite/Formula	Source/Characteristics	Model type (zebrafish)	Core effects	Mechanism of action	References
TCM active Metabolites/Microbial metabolites	*Semen Ziziphi Spinosae*	*Ziziphus jujuba* Mill. var. *spinosa* (Bunge) Hu ex H.F.Chow [Rhamnaceae; Ziziphi spinosae semen]	Light stimulation	Reduces arousal activity and duration; promotes resting states	Regulates expression of neurotransmitter receptors	Xia et al. (2019)
	Ginsenoside Rg1	TCM-derived active metabolite from *Panax ginseng*	72 h LED light sleep deprivation model	Improves behavior; alleviates brain damage; increases oxidative stress-related enzyme activity	Mediated by Bcl-2/Bax/caspase-3 apoptotic signaling pathway	Lu et al. (2023)
	Probiotic-fermented *Gastrodia elata* Blume (GE)	Fermented product of *Gastrodia elata* Blume [Orchidaceae; Gastrodiae rhizoma]	PTZ-induced insomnia	Ameliorates sleep disturbances; outperforms unfermented GE	Maintains serotonin balance via gut microbiota and amino acid metabolism; regulates neuroactive ligand-receptor interactions and DNA repair	Zhang X. et al. (2025)
	*Schisandra chinensis*	TCM with neuroactive properties	Behavioral evaluation model	Exhibits neuroactive properties	Linked to both serotonin and dopamine pathways	Wang et al. (2018)
	*Ganoderma leucocontextum* extracts	Novel species endemic to Qinghai-Tibet Plateau (medicinal fungus)	Caffeine-induced insomnia	Reduces locomotor activity	Synergistic efficacy via “multi-component, multi-target, multi-pathway” mode	Zhao et al. (2025)
TCM prescriptions	Anmeidan	TCM prescription	Induced by caffeine and LED light	Repairs circadian rhythms; regulates energy metabolism; increases resting zebrafish count; reduces hyperactivity; alleviates neuronal damage	Downregulates clock gene expression; enhances ATPase activity; modulates OXA/P38 MAPK/ERK1/2 signaling pathway	Wang Y. et al., 2022; Ye et al., 2024
	Huanglian Wendan decoction (HWD)	Classic TCM prescription	PCPA-induced insomnia	Exerts anti-insomnia effects; relieves hyperactive movement	Activates GABA_A_ receptors to enhance GABA-induced currents in specific cells	Li L. et al. (2024)
	Chaihu Longgu Muli decoction (CLMD)	Classic TCM prescription	Insomnia and anxiety comorbidity model (caffeine-induced)	Improves sleep-wake behaviors; reduces anxiety-related behaviors	Regulates neurotransmission and inflammatory pathways; targets IL6 and TNF	Fan et al. (2025)

### 3.6 Applications in other neurological diseases

Zebrafish models have been widely used to study TCM interventions for depression, spinal cord injury, and nerve repair. Due to their unique biological characteristics and experimental advantages, zebrafish have established an important position in antidepressant research of TCM.

In depression research, the chronic unpredicted mild stress (CUMS) and reserpine-induced models, combined with behavioral assays, provide reliable platforms for evaluating TCM efficacy (Zhang et al., 2018; Zhang et al., 2021). *Platycladus orientalis* (L.) Franco [Cupressaceae; Platycladi cacumen/Platycladi semen] seed extract (S4) reverses depressive phenotypes by regulating monoamine metabolism (Yan et al., 2022). Gastrodin (from *Gastrodia elata* Blume [Orchidaceae; Gastrodiae rhizoma]) activates Nrf2 signaling, promotes Arg-1^+^ microglial phenotype conversion, and inhibits IL-1 and TNF-α release, alleviating LPS-induced neuroinflammation and anxiety-like behaviors (Zhang et al., 2023). *Panax ginseng* C.A.Mey. [Araliaceae; Ginseng radix et rhizoma], *Crocus sativus* L. [Iridaceae; Croci stigma], and *Hypericum perforatum* L. [Hypericaceae; Hyperici herba] extracts show dose-dependent sedative effects, validating zebrafish as a screening platform for neuroactive TCM metabolites (Wang T. et al., 2025). Jia Wei Xiao Yao (JWXY) capsule demonstrates superior efficacy to sertraline in reserpine-induced depression models (Zhang et al., 2018). Anshen Buxin Six Pills (ASBX) improves behavioral indicators, reduces neural damage, and elevates 5-HT and NE levels, establishing a high-throughput phenotypic screening framework (Liu et al., 2021).

For spinal cord injury and nerve repair, zebrafish models using mechanical transection or laser ablation enable dynamic observation of axonal regeneration and glial scar formation (Zhang et al., 2021). Injury-induced serotonergic neuron subpopulations contribute to axonal regrowth and functional recovery (Huang et al., 2021). Astragaloside IV (from *Astragalus mongholicus* Bunge [Fabaceae; Astragali radix]) enhances neural stem cell proliferation after injury (Lu et al., 2021). *Panax notoginseng* (Burkill) F.H.Chen [Araliaceae; Notoginseng radix et rhizoma] saponins (PNS) activate Wnt/β-catenin signaling, accelerating axonal regeneration and improving motor function (Hong et al., 2008). Gastrodin promotes myelination via PI3K/AKT/mTOR signaling, demonstrating reparative potential for demyelinating lesions (Shi et al., 2025). Naomaitai Capsule (a TCM compound prescription) promotes nerve regeneration in peripheral motor neuron injury models (Zhong et al., 2021).

## 4 Common characteristics of TCM active metabolites and strategies for efficacy and toxicity control in zebrafish models

### 4.1 Common characteristics of TCM active metabolites

Based on the literature reviewed, TCM active metabolites with neuroprotective effects in zebrafish share several common properties.

1. Low molecular weight. Most have molecular weights below 600 Da (e.g., berberine 336 Da, gastrodin 286 Da, puerarin 416 Da), facilitating passive diffusion across biological membranes, including the blood-brain barrier (BBB). The zebrafish BBB becomes functional by approximately 3 dpf and exhibits conserved structural and functional characteristics with the mammalian BBB, enabling reliable assessment of compound permeability (Li et al., 2017; Kotova et al., 2025).
2. Polyphenolic or flavonoid structures. Many neuroprotective TCM metabolites are flavonoids, phenolic acids, or other polyphenols, which confer potent antioxidant activity, scavenge reactive oxygen species (ROS), and upregulate endogenous defense systems such as Nrf2/HO-1. Similar properties are observed in Ayurvedic medicinal plants, which are also rich in flavonoids, lignans, sterols, tannins, and alkaloids (Chakraborty et al., 2025).
3. Multi-target modulation. Unlike single-target synthetic drugs, TCM active metabolites interact with multiple signaling pathways, including anti-inflammatory (NF-κB, MAPK, NLRP3), anti-apoptotic (PI3K/Akt, Bcl-2/Bax/caspase-3), antioxidant (Nrf2/HO-1, SOD, CAT, GPx), neurotransmitter regulation (AChE inhibition, monoamine modulation), and autophagy (LC3-II/I, p62). This multi-target characteristic aligns with TCM’s holistic philosophy and is consistently observed in zebrafish models (Abidar et al., 2025).
4. Adequate solubility for immersion administration. Most studies administer compounds via immersion (bath exposure), requiring sufficient solubility in aqueous medium or in DMSO at concentrations ≤0.1–0.5% (v/v) to avoid solvent toxicity. Many TCM metabolites, especially glycosides and polar compounds, meet this requirement.
5. Dose-dependent hormetic effects. Many TCM metabolites exhibit biphasic dose-response relationships (hormesis): low doses are beneficial, while high doses may be ineffective or toxic. For example, berberine exerts neuroprotection at low concentrations but cytotoxicity at higher doses via PI3K/AKT/Bcl-2 and Nrf2/HO-1 pathways (Zhang et al., 2017b). This necessitates careful dose optimization, as demonstrated for TCM botanical drug extracts (Wang T. et al., 2025).
6. BBB permeability or peripheral modulation. To exert direct neuroprotective effects, metabolites must penetrate the BBB or modulate peripheral pathways that indirectly affect the CNS. The zebrafish BBB is a validated model for assessing natural product delivery across the BBB, offering advantages for high-throughput screening and real-time imaging (Li et al., 2017). Many studied metabolites effectively cross the BBB and accumulate in brain tissue.

### 4.2 Strategies for efficacy control and toxicity reduction

Rigorous experimental design is essential for reliable efficacy assessment and minimization of metabolite-induced toxicity.

#### 4.2.1 Efficacy control strategies

1. Dose-response curves. Multiple concentrations (typically 3-6 doses spanning at least one order of magnitude) are tested to establish dose-response relationships. The lowest effective concentration (LEC) and half-maximal effective concentration (EC_50_) are calculated. Dose-dependent behavioral responses have been systematically characterized for TCM botanical drugs (Wang T. et al., 2025).
2. Positive controls. Validated positive drugs (e.g., donepezil for AD, L-DOPA for PD, valproic acid for epilepsy, melatonin for insomnia) are included to benchmark efficacy at clinically relevant or previously validated concentrations.
3. Multiple efficacy endpoints. Neuroprotective effects are assessed using complementary endpoints: behavioral assays (locomotion, memory, seizure scoring, sleep/wake tracking), molecular assays (qPCR, Western blot), biochemical assays (oxidative stress markers, neurotransmitter levels), and histological analyses (immunostaining, apoptosis detection). Integration of multiple endpoints provides robust efficacy assessment.
4. Statistical rigor. Adequate sample sizes (n ≥ 10-30 per group), randomization, blinded assessment where feasible, and appropriate statistical tests (ANOVA with *post hoc*, two-way ANOVA) are used. Automated behavioral tracking and image analysis enhance objectivity and reproducibility.

#### 4.2.2 Toxicity control and reduction strategies

1. Determination of MTC and LC_50_. Acute toxicity is assessed before efficacy studies to establish safe concentration ranges. The LC_50_ (50% lethal concentration) and MTC (maximum tolerated concentration) are determined via dose-finding experiments. For example, LC_50_ values for *Penthorum chinense* Pursh [Penthoraceae] extract and gallic acid in zebrafish embryos were 237.0 mg/L and 328.4 mg/L, respectively (Liu K. et al., 2025). Typically, concentrations below MTC (1/3 to 1/10 of LC_50_) are selected for efficacy studies.
2. Monitoring morphological and developmental toxicity. Embryos and larvae are examined for mortality, hatching rate, morphological abnormalities (edema, spinal curvature, yolk sac malformation, tail deformation, craniofacial defects), heart rate changes, pigmentation, and body length. High concentrations of *P. chinense* extract induce cardiac malformations and swim bladder abnormalities, underscoring the need for comprehensive developmental toxicity assessment (Liu K. et al., 2025).
3. Organ-specific toxicity. Hepatotoxicity (liver morphology, gene expression, histopathology), cardiotoxicity (heart rate, rhythm, circulation, pericardial edema), neurotoxicity (behavior, neuronal morphology), and renal toxicity (glomerular morphology, edema) are evaluated. Zebrafish are increasingly used for high-throughput drug toxicity screening (Li X. et al., 2025).
4. Developmental stage selection. Larvae at 5-7 dpf are most common due to optical transparency and functional BBB, but sensitivity may differ from adults. Pilot toxicity studies use the same stage as efficacy experiments.
5. Vehicle and solvent controls. Solvent controls (e.g., DMSO, ethanol, methanol) at the same concentration as in treatment groups are included to distinguish metabolite-specific effects. Final DMSO concentration should not exceed 0.1%–0.5% (v/v).
6. Therapeutic window determination. The therapeutic window is the ratio of NOAEL (no-observed-adverse-effect level) to MEC (minimum effective concentration). A wider window indicates a safer profile. Compounds with narrow windows may be excluded or optimized via formulation.
7. Formulation optimization. For poorly soluble or toxic compounds, advanced formulations improve bioavailability and brain delivery while reducing toxicity, including nanocrystals (ginkgolide B, puerarin), nanoparticles (PEG-PCL), and liposomes (curcumin) (Liu et al., 2020; Zhao et al., 2020; Xiong et al., 2019).
8. Range-finding experiments. Prior to full efficacy studies, a wide concentration range (e.g., 0.1–1,000 μM or 0.1–1,000 μg/mL) is tested to identify the window of neuroprotective activity without overt toxicity. Selected concentrations for efficacy studies are within the safe range (≤MTC) and span the expected EC_50_.

## 5 Prospects for clinical translation of the zebrafish model in TCM neuroprotection research

The zebrafish model, characterized by optical transparency, rapid development, high-throughput screening compatibility, and neural pathways that are evolutionarily conserved with humans, serves as a valuable bridge for translating TCM-based neuroprotective strategies into clinical practice. Its primary applications span three interconnected dimensions: personalized medicine, precision medicine, and the optimization of clinical trial quality. Together, these approaches offer an innovative framework for the standardized application of TCM in neurological disorders.

In the context of personalized medicine, the zebrafish xenograft model (zAvatar) enables the rapid establishment of patient-specific disease models through the transplantation of patient-derived neurons or neural tissues (Costa et al., 2024). When integrated with the TCM principle of “syndrome differentiation and treatment,” this platform facilitates individualized efficacy evaluation and regimen optimization for TCM metabolites. In oncology, this technology has demonstrated a concordance rate exceeding 80% with clinical responses, providing a valuable technical reference for precision-guided TCM interventions in neurodegenerative diseases such as Alzheimer’s and Parkinson’s disease (Barbosa et al., 2025).

**FIGURE 3:**
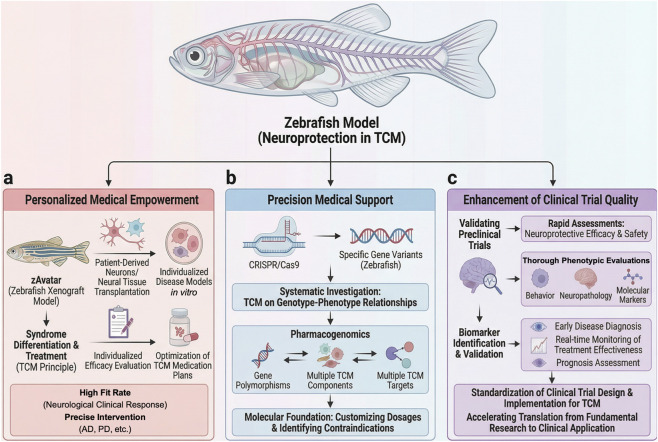
Translational value of zebrafish models in advancing neuroprotective TCM. **(a)** Personalized Medical Empowerment: Zebrafish avatar models (xenografts) and patient-derived neuron transplantation generate individualized *in vitro* disease models, which are combined with TCM syndrome differentiation to deliver precise interventions for conditions such as AD and PD, optimize medication plans, and achieve high clinical response fit rates in neurology. **(b)** Precision Medical Support: CRISPR/Cas9-mediated introduction of human gene variants into zebrafish enables systematic investigation of TCM-modulated genotype-phenotype relationships. Pharmacogenomic studies further explore interactions between gene polymorphisms, multi-component TCM formulas, and therapeutic targets, establishing a molecular basis for dosage customization and contraindication identification. **(c)** Enhancement of Clinical Trial Quality: Zebrafish models validate preclinical trials via rapid efficacy/safety assessments and comprehensive phenotypic evaluations (behavior, neuropathology, molecular markers). This facilitates biomarker discovery and validation to support early diagnosis, real-time treatment monitoring, and prognosis assessment, driving the standardization of TCM clinical trial design and accelerating bench-to-bedside translation.

In the field of precision medicine, zebrafish lines harboring specific genetic variants—generated using CRISPR/Cas9 and other gene-editing tools—enable systematic investigation of how TCM modulates genotype-phenotype relationships (Siddiqui et al., 2025). Concurrently, pharmacogenomic studies can elucidate how gene polymorphisms influence the complex interactions among multiple metabolites and targets within TCM formulas (Cornet et al., 2018). These lines of inquiry provide a molecular basis for dose customization and identification of contraindications in TCM therapy (Figure 3). Furthermore, zebrafish models are instrumental in preclinical validation, allowing rapid assessment of neuroprotective efficacy and safety through comprehensive phenotyping encompassing behavior, neuropathology, and molecular biomarkers (Liu et al., 2025a). In addition, the use of zebrafish models for biomarker discovery and validation can generate objective metrics for early disease diagnosis, real-time treatment monitoring, and prognosis evaluation in neurological disorders (Elsaid et al., 2023). Collectively, these efforts not only improve the standardization of clinical trial design and implementation for TCM but also accelerate the translation of neuroprotective formulations from basic research to clinical application (Ngu et al., 2025).

## 6 Challenges and prospects

Although zebrafish models offer considerable potential for investigating neuroprotective mechanisms of TCM, several challenges remain to be addressed. These challenges primarily concern the standardization of experimental protocols, the translation of findings across species, and the adaptation to the inherent complexity of TCM systems.

A major limitation is the lack of standardized protocols for establishing and validating zebrafish disease models across different laboratories, which often leads to poor reproducibility and comparability of results. Furthermore, the diversity in data formats, analytical methods, and reporting standards generated by high-throughput technologies underscore the urgent need for a unified data management framework (Kawaguchi and Nakanishi, 2023). Due to evolutionary divergence between zebrafish and humans, certain research findings may not be directly translatable to clinical settings, highlighting the necessity for rigorous cross-species validation (Burgess and Burton, 2023). At the practical level, most current zebrafish disease models mimic only single pathological features, which limits their ability to recapitulate the multifactorial and progressive nature of human neurological disorders. In addition, the intrinsic complexity of TCM—characterized by multi-component, multi-target, and multi-pathway interactions—poses challenges for the relatively simplified framework of zebrafish models. Consequently, innovative research strategies are urgently needed to comprehensively dissect the mechanisms of TCM action (Saleem and Kannan, 2018).

Looking forward, the continued development of zebrafish models in TCM neuroprotection research will benefit from interdisciplinary collaboration and technological innovation (Liu et al., 2025b). A key research direction involves the integration of multi-omics approaches—including genomics, transcriptomics, proteomics, and metabolomics—to systematically elucidate the molecular mechanisms underlying TCM (Wen et al., 2025; Zhao et al., 2024). The broader application of artificial intelligence (AI) technologies is expected to significantly improve the efficiency and accuracy of data analysis (Mahmud et al., 2021). By employing machine learning, deep learning, and other AI methodologies, researchers may uncover fundamental biological principles, facilitate the identification of bioactive TCM metabolites, and clarify their mechanisms of action (Olawade et al., 2025). Moreover, progress toward clinical translation will be accelerated through the establishment of standardized disease models, the development of more complex models that better reflect human pathology, and the refinement of cross-species validation systems (Cassar et al., 2019). These collective efforts aim to bridge the gap between basic research and clinical practice, thereby providing a robust scientific foundation for the precise application of TCM in the treatment of neurological disorders (Burton and Burgess, 2022).

## 7 Conclusion

In summary, the zebrafish model offers a useful and complementary platform for investigating the neuroprotective effects of TCM in various neurological disorders. This review has summarized its applications in Alzheimer’s disease, Parkinson’s disease, cerebral ischemia, epilepsy, insomnia, depression, and spinal cord injury. The available evidence suggests that TCM exerts multi-target actions, including anti-oxidative stress, anti-neuroinflammation, anti-apoptosis, neurotransmitter modulation, neurogenesis promotion, and vascular protection. The integration of behavioral assays with automated and deep-learning technologies has improved the throughput and objectivity of pharmacodynamic evaluations, thereby providing technical support for TCM modernization.

Practical applications of the zebrafish model in TCM research currently include: (i) high-throughput screening of active metabolites and botanical drug extracts; (ii) rapid *in vivo* validation of multi-target mechanisms; (iii) real-time imaging of neurovascular and regenerative processes; and (iv) preliminary safety and toxicity assessment. These features make zebrafish a cost-effective and ethically acceptable complement to rodent models, particularly during early-stage drug discovery.

Nevertheless, several limitations of the current review and the model itself should be acknowledged. First, despite genetic homology, zebrafish lack a six-layered neocortex and possess a substantially simpler brain architecture, which limits the recapitulation of higher-order cognitive deficits and complex human neuropathologies. Second, differences in drug metabolism, blood-brain barrier permeability, and pharmacokinetics between zebrafish and mammals necessitate cross-species validation. Third, most existing zebrafish disease models are acute or subacute, making chronic neurodegenerative processes difficult to reproduce. Fourth, behavioral endpoints—such as locomotor activity, seizure-like events, and sleep patterns—are quantifiable but may not fully mirror human clinical symptoms. Fifth, the remarkable regenerative capacity of the zebrafish nervous system may lead to underestimation of injury severity and overestimation of therapeutic effects relative to humans. Sixth, many TCM studies using zebrafish lack standardized protocols (e.g., induction methods, dosing regimens, outcome measures), resulting in limited inter-laboratory reproducibility.

Future perspectives may focus on: (i) establishing standardized and validated zebrafish disease models with clearly defined endpoints; (ii) developing chronic and comorbid models that better mimic human disease progression; (iii) integrating multi-omics, CRISPR-based screening, and artificial intelligence to dissect mechanisms of action and identify novel therapeutic targets; (iv) combining zebrafish phenotypic screening with mammalian validation pipelines to improve translational predictability; (v) promoting open data and protocol sharing to enhance reproducibility; and (vi) developing zebrafish-based strategies to evaluate TCM formula compatibility and synergistic effects. Addressing these challenges will help position the zebrafish model as a more reliable and complementary tool for TCM neuroprotection research and facilitate its thoughtful translation toward clinical applications.

## Glossary

5-HT: 5-Hydroxytryptamine
Aβ: β-amyloid
ABCA1: ATP-Binding Cassette Transporter A1
AChE: Acetylcholinesterase
AD: Alzheimer’s disease
AMPA: α-Amino-3-Hydroxy-5-Methyl-4-Isoxazolepropionic Acid
AMPK: Adenosine Monophosphate-Activated Protein Kinase
ANP: Angong Niuhuang Pill
APP: Amyloid Precursor Protein
ASBX: Anshen Buxin Six Pills
ASDs: Autism Spectrum Disorders
BECCs V: Combinatorial Component V of Angong Niuhuang Pill
BBR: Berberine
BBR-D1: Berberine Derivative D1
BBRP: Fluorescently Labeled Berberine Derivative
BC: Berberine Chloride
BChE: Butyrylcholinesterase
BCS: Biopharmaceutics Classification System
BLU: Ethanol extract of *Arisaema heterophyllum*Blume
Bcl-2/Bax: B-Cell Lymphoma 2/Bcl-2-Associated X Protein
CAT: Catalase
CGA: Chlorogenic Acid
CLMD: Chaihu Longgu Muli Decoction
CNS: Central Nervous System
COLA: *Calendula officinalis* L. Extract
C. *elegans*: *Caenorhabditis elegans*
CUMS: Chronic Unpredicted Mild Stress
CT: *Cistanche tubulosa* (Schenk) Wight
DALYs: Disability-Adjusted Life Years
DA: Dopaminergic
DGSN: Dang-Gui-Si-Ni Decoction
DS: *Salvia miltiorrhiza* (Danshen)
ECJ: Ethanol Extract of *Caragana jubata*
EGFR: Epidermal Growth Factor Receptor
EGFP: Enhanced Green Fluorescent Protein
EKP: Ethylketopentenoate
EUMF: *Eucommia ulmoides* Olive Male Flower Extract
FAK: Focal Adhesion Kinase
FRET: Fluorescence Resonance Energy Transfer
GABA: γ-Aminobutyric Acid
**GABA**_**A**_: γ-Aminobutyric Acid Type A Receptor
GB-NCs: Ginkgolide B Nanocrystals
GF1: Ginsenoside F1
GHI: Guhong Injection
GL: Glycyrrhizin
GSK3β: Glycogen Synthase Kinase 3β
GSDMD: Gasdermin D
HA-CA: *Centella asiatica* hydroalcoholic extract
HMGB1: High-Mobility Group Box 1
Hcrt: Hypocretin/Orexin
HcrtR: Hypocretin/Orexin Receptor
HES: Hesperidin
HMOX1: Heme Oxygenase 1
HWD: Huanglian Wendan Decoction
HD: Huntington’s Disease
IGF-1/IGF1R: Insulin-Like Growth Factor 1/Receptor
IF: Intermittent Fasting
iNOS: Inducible Nitric Oxide Synthase
IL-1β: Interleukin-1β
IL6: Interleukin-6
JNK: c-Jun N-Terminal Kinase
JWXY: Jia Wei Xiao Yao Capsule
*kdrl*: Kinase Insert Domain Receptor-Like
*kcnd2*: Potassium Voltage-Gated Channel Subfamily D Member 2
*kcnv1*: Potassium Voltage-Gated Channel Subfamily V Member 1
LPS: Lipopolysaccharide
*LRRK2*: Leucine-Rich Repeat Kinase 2
LC-MS: Liquid Chromatography-Mass Spectrometry
LFP: Local Field Potential
MAO-B: Monoamine Oxidase B
MAPK: Mitogen-Activated Protein Kinase
MGF: Mangiferin
MPTP: 1-Methyl-4-phenyl-1,2,3,6-tetrahydropyridine
**MPP** ^**+**^: 1-Methyl-4-phenylpyridinium Ion
NF-κB: Nuclear Factor-κB
Nrf2/HO-1: Nuclear Factor Erythroid 2-Related Factor 2/Heme Oxygenase-1
NLRP3: NOD-Like Receptor Pyrin Domain-Containing 3
NO: Nitric Oxide
NOT: Notopterol
NPs: Nanoparticles
Naringenin: Naringenin
OST: Osthole
OP: Osteoporosis
PCPA: Parachloro Phenylacetic Acid
PCRR: *Polygoni Cuspidati* Rhizoma et Radix extract
PD: Parkinson’s disease
PEG-PCL: Polyethylene Glycol-Polycaprolactone
PHZ: Phenylhydrazine
PI3K/Akt: Phosphatidylinositol 3-Kinase/Protein Kinase B
PINK1: PTEN-Induced Kinase 1
PNS: Panax Notoginseng Saponin Extract
PRE: *Polygalae Radix* extracts
PTS: Panaxatriol Saponins/Protoparaxotriol Saponins
PTZ: Pentylenetetrazol
PU-NCs: Puerarin Nanocrystals
PU: Puerarin
QLC: Qilong Capsule
QC: *Rubia cordifolia* extract
ROS: Reactive Oxygen Species
SA-NCs: Schisantherin A Nanocrystals
SA: Schisantherin A
SCI: Spinal Cord Injury
SIRT3: Sirtuin 3
SNCA: α-Synuclein Gene
SOD: Superoxide Dismutase
STX1B: Syntaxin 1B
SW: Sanwujiao Granule
SQ: *Panax notoginseng* (Sanqi)
SLC7A11: Solute Carrier Family 7 Member 11
SvMEs: *S. vaninii* mycelium extracts
TLR4: Toll-Like Receptor 4
TNF: Tumor Necrosis Factor
TTKFY: Tongtian Oral Liquid
TRPV1: Transient Receptor Potential Vanilloid 1
TRPM8: Transient Receptor Potential Melastatin 8
TCM: Traditional Chinese Medicine
Tauopathies: Tau-related Pathologies
VEGFR2: Vascular Endothelial Growth Factor Receptor 2
VEGF: Vascular Endothelial Growth Factor
VRI: Vascular Endothelial Growth Factor Receptor Inhibitor
zAvatar: Zebrafish Xenograft Model
UPLC-QTOF-MS: Ultra-High Performance Liquid Chromatography-Quadrupole Time of Flight-Mass Spectrometry
HPLC-Q-TOF-MS/MS: High-Performance Liquid Chromatography-Quadrupole-Time of Flight-Tandem Mass Spectrometry
Ge/PLA/AuNCs: Gelatin/PLA/Gold Nanocomposites
AMPK/SIRT1/FOXO3: Adenosine Monophosphate-Activated Protein Kinase/Sirtuin 1/Forkhead Box O3
PI3K/AKT/mTOR: Phosphatidylinositol 3-Kinase/Protein Kinase B/Mammalian Target of Rapamycin
OXA/P38 MAPK/ERK1/2: Orexin/P38 Mitogen-Activated Protein Kinase/Extracellular Signal-Regulated Kinase 1/2
HMGB1-TLR4-NF-κB: High-Mobility Group Box 1-Toll-Like Receptor 4-Nuclear Factor-κB
NLRP3/caspase -1/GSDMD: NOD-Like Receptor Pyrin Domain-Containing 3/Cysteinyl Aspartate-Specific Protease 1/Gasdermin D
SLC7A11/GPX4: Solute Carrier Family 7 Member 11/Glutathione Peroxidase 4
VEGF/PI3K/Akt/MAPK: Vascular Endothelial Growth Factor/Phosphatidylinositol 3-Kinase/Protein Kinase B/Mitogen-Activated Protein Kinase
JNK/p38 MAPK: c-Jun N-Terminal Kinase/p38 Mitogen-Activated Protein Kinase
Akt/Gsk3β: Protein Kinase B/Glycogen Synthase Kinase 3β
Nrf2-ARE: Nuclear Factor Erythroid 2-Related Factor 2-Antioxidant Response Element
Bcl-2/Bax/caspase-3: B-Cell Lymphoma 2/Bcl-2-Associated X Protein/Cysteinyl Aspartate-Specific Protease 3
*casp3/casp9*: Cysteinyl Aspartate-Specific Protease 3/9
*polg*: DNA Polymerase Gamma
*pck1*: Phosphoenolpyruvate Carboxykinase 1
*ubr5*: Ubiquitin-Protein Ligase E3 Component N-Recognin 5
*wnt8b*: Wnt Family Member 8B
*arfgef1*: ADP-Ribosylation Factor Guanine Nucleotide Exchange Factor 1
*dat*: Dopamine Transporter
*th*: Tyrosine Hydroxylase
*α-syn*: α-Synuclein
*lc3b*: Microtubule-Associated Protein 1 Light Chain 3 Beta
*atp13a*: ATPase 13A
*dnajc6*: DnaJ Heat Shock Protein Family (Hsp40) Member C6
*uchl1*: Ubiquitin C-Terminal Hydrolase L1
*vps35*: Vacuolar Protein Sorting-Associated Protein 35
*Anxa1c*: Annexin A1c
*Ptger1a*: Prostaglandin E Receptor 1a
*c-fos*: FBJ Murine Osteosarcoma Viral Oncogene Homolog
EdU: 5-Ethynyl-2'-Deoxyuridine
AMPA receptors: α-Amino-3-Hydroxy-5-Methyl-4-Isoxazolepropionic Acid Receptors
glutamic acid decarboxylase: Glutamic Acid Decarboxylase
AdKuz2: Kunitz-Like Peptide from *Acropora digitifera*
CRISPR/Cas9: Clustered Regularly Interspaced Short Palindromic Repeats/CRISPR-Associated Protein 9
